# A multi-source domain annotation pipeline for quantitative metagenomic and metatranscriptomic functional profiling

**DOI:** 10.1186/s40168-018-0532-2

**Published:** 2018-08-28

**Authors:** Ari Ugarte, Riccardo Vicedomini, Juliana Bernardes, Alessandra Carbone

**Affiliations:** 1 Sorbonne Université, UPMC-Univ P6, CNRS, IBPS, Laboratoire de Biologie Computationnelle et Quantitative - UMR 7238, 4 Place Jussieu, Paris, 75005 France; 2 Sorbonne Université, UPMC-Univ P6, CNRS, Institut des Sciences du Calcul et des Donnees, 4 Place Jussieu, Paris, 75005 France; 3 Institut Universitaire de France, Paris, 75005 France

**Keywords:** Domain annotation, Metagenomic, Metatranscriptomic, Functional annotation, Probabilistic model, Environment, Motif

## Abstract

**Background:**

Biochemical and regulatory pathways have until recently been thought and modelled within one cell type, one organism and one species. This vision is being dramatically changed by the advent of whole microbiome sequencing studies, revealing the role of symbiotic microbial populations in fundamental biochemical functions. The new landscape we face requires the reconstruction of biochemical and regulatory pathways at the community level in a given environment. In order to understand how environmental factors affect the genetic material and the dynamics of the expression from one environment to another, we want to evaluate the quantity of gene protein sequences or transcripts associated to a given pathway by precisely estimating the abundance of protein domains, their weak presence or absence in environmental samples.

**Results:**

MetaCLADE is a novel profile-based domain annotation pipeline based on a multi-source domain annotation strategy. It applies directly to reads and improves identification of the catalog of functions in microbiomes. MetaCLADE is applied to simulated data and to more than ten metagenomic and metatranscriptomic datasets from different environments where it outperforms InterProScan in the number of annotated domains. It is compared to the state-of-the-art non-profile-based and profile-based methods, UProC and HMM-GRASPx, showing complementary predictions to UProC. A combination of MetaCLADE and UProC improves even further the functional annotation of environmental samples.

**Conclusions:**

Learning about the functional activity of environmental microbial communities is a crucial step to understand microbial interactions and large-scale environmental impact. MetaCLADE has been explicitly designed for metagenomic and metatranscriptomic data and allows for the discovery of patterns in divergent sequences, thanks to its multi-source strategy. MetaCLADE highly improves current domain annotation methods and reaches a fine degree of accuracy in annotation of very different environments such as soil and marine ecosystems, ancient metagenomes and human tissues.

**Electronic supplementary material:**

The online version of this article (10.1186/s40168-018-0532-2) contains supplementary material, which is available to authorized users.

## Background

Ecosystem changes are often correlated with the presence of new communities disturbing their stability by importing new metabolic activities [1–4]. Very often, such communities, their functional behaviour and their mutual interactions are hard to identify and analyse [5–9]. Unraveling their structure and determining what they functionally do is crucial for understanding their metabolic dynamics and activities.

Computational studies improving the detection of the functional preferences of environmental communities are important for gaining insight into ecosystem changes [10–15]. Ideally, they shall quantitatively relate genetic information with environmental factors in order to understand how these factors affect the genetic material and the dynamics of the expression from one environment to another, from one community to another. Therefore, they demand the development of appropriate tools to zoom in metabolic activities and to compare environments in detail being as precise as possible in evaluating the quantity of genetic material (gene protein sequences or transcripts) associated to a given function.

Over the past years, a lot of effort has been devoted to the creation of integrated systems for the computational analysis of metagenomic (MG) and metatranscriptomic (MT) datasets. Several pipelines conducting data pre-processing, assembly, taxonomic characterisation, gene finding, protein-coding gene annotation and pathway reconstruction, such as MinPath [16], ShotgunFunctionalizeR [17], CAMERA [18], CoMet [19], IMG/M [20, 21], MetaPath [22], PICRUSt [23], Genometa [24], MetaPathway [25], COGNIZER [26], MG-RAST [27, 28], MEGAN [29] and MOCAT2 [30], have been proposed [31]. For functional characterisation, protein gene annotation remains a fundamentally difficult task that still needs to be improved in order to better understand the billions of sequences that remain functionally unannotated [32, 33]. One difficulty comes from the fact that most environmental coding sequences present no or very weak similarity with known sequences and that many of them might be new genes with novel functions. In practice, they often do not match reference databases or they match with very low significance scores [34], leading to a poor functional annotation [32]. A second difficulty is that environmental coding sequences are fragmented and annotation of partial information becomes harder due to a much reduced sequence length. In this respect, since protein-coding sequences might be too long compared to reads, in environmental sequence classification, one can either realise a simultaneous alignment and assembly of reads using reference proteins or probabilistic protein sequence profiles [35–37] hoping to improve the sensitivity to detect significant matches, or can focus on annotating protein domains directly on sequencing reads [38]. Indeed, domains are functional units, much shorter than proteins: even though their sizes vary from a few tens up to several hundreds of amino acids, 90% of the known domains are smaller than 200 aa with mean size of 100 aa [39–41]. Despite their short length, they are sufficiently precise to inform us about the potential functional activity of the communities. In particular, direct read annotation will become increasingly important in the future, due to its contribution in the design of computationally efficient and precise assembly algorithms [42]. With the production of larger and larger MG/MT datasets and the exploration of new environments (possibly gathering many unknown species), contig reconstruction might become even more challenging if realised without the help of domain annotation.

Here, we introduce MetaCLADE, a new generation method for the annotation of protein domains in MG/MT reads. MetaCLADE uses a multi-source annotation strategy [43], where each known domain is represented by a few hundred probabilistic models and an intelligent algorithmic strategy filters the high number of hits produced by the models, retaining only the most reliable ones. These models, called clade-centered models (CCMs), span regions of the protein sequence space that are usually not well represented in a model based on a global sequence consensus (SCM) [44–47]. They might highlight motifs, structural or physico-chemical properties characteristic of divergent homologous sequences. Hence, if a domain is associated to many divergent homologs, CCMs are expected to describe properties that could be missed by the SCM representing a global consensus. For this reason, CCMs should help in finding diverged homologous sequences in species that might be phylogenetically distant.

The great improvement in annotation obtained with the multi-source strategy compared to the mono-source strategy, employed by the two most commonly used annotation tools HMMer [47] and HHblits [48, 49], was proven for CLADE in [43, 50] for genomes and, more generally, for datasets of complete coding sequences. Many different validation tests have been developed in [43], where, in particular, it has been shown that many of the annotations realised with CLADE based on domains present in Pfam24 and considered false positives by Pfam24 were finally validated by the Pfam27 release, this augmenting the confidence on CLADE annotation of protein sequences. With the introduction of MetaCLADE, however, we push forward this idea and we show that CCMs can also be used to successfully annotate fragmented coding sequences in MG/MT datasets, where domain divergence and species variability might be very large. Under the hypothesis that the most populated functional classes define community preferences, the high-quality quantification of domains in reads reached with MetaCLADE allows us to infer domain functional classification and the functional importance of species in a community. Differences in domain counts for specific functional classes can also be used to compare and characterise environments.

For the annotation of MG/MT sequences, the main improvement of MetaCLADE over CLADE lies in the manner clade-centered models are handled. In fact, MetaCLADE employs neither the machine-learning algorithm for domain annotation nor the algorithm constructing the most likely protein domain architecture, characterising the two main algorithmic steps in CLADE. Due to the traits of MG/MT reads compared to full protein sequences (e.g., the very short length), the design of a specific computational method was demanded. Hence, in order to annotate fragmented domains and evaluate much shorter hits, MetaCLADE was designed around two main contributions. First, it introduces a novel definition for a domain-specific bi-dimensional gathering threshold based on a probability space constructed using a naive Bayes classifier. Second, it simplifies the hit selection so that domain annotation would be less sensitive to sequencing errors and hit length.

In order to show how MetaCLADE performs with respect to known domain annotation methods, we realised multiple comparative analyses of MG/MT datasets and demonstrated that MetaCLADE improves domain annotation and provides an improved resolution of the functional activity of a community, while clarifying preferences and missing functional features. We compared MetaCLADE against InterProScan [51, 52], a tool that combines different protein signature recognition methods and different domain databases; UProC [38], a fast and sensitive algorithm that applies directly to raw reads; and the profile-based assembler HMM-GRASPx [37]. These are either new or most commonly used metagenomic annotation tools and, as they are based on Pfam, we can fairly compare MetaCLADE to them. We used several datasets, based on either real or simulated MG/MT sequences, with different characteristics, such as read length (100 bp versus 200 bp) and non-uniform species relative abundance. The simulated datasets have been generated either from known complete genomes or, more realistically, from MG datasets.

## Results

Protein domains found in short MG and MT reads can be used as precise markers of the functional activity of an environment. We show that MetaCLADE highly improves current annotation methods and reaches a very fine degree of accuracy in annotation.

**Fig. 1:**
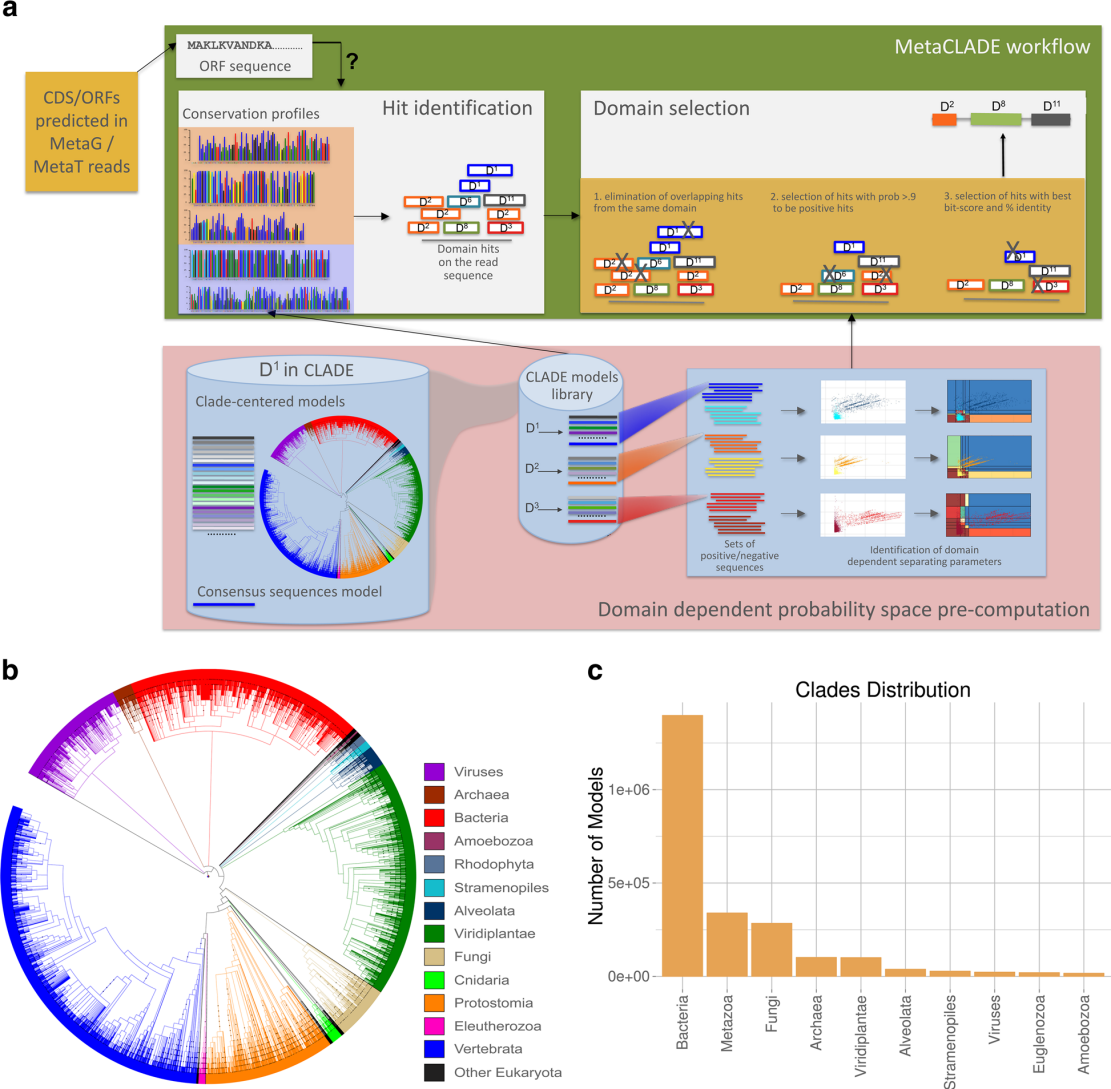
The MetaCLADE workflow. **a** The MetaCLADE workflow is described in the rectangular green box: the two MetaCLADE main steps are illustrated in white boxes. MetaCLADE input data is constituted by (i) a set of reads to be annotated where ORFs have been already identified and (ii) the CLADE model library. The CLADE model library is used to identify all domain hits for a ORF. The large set of identified hits is then combined with gathering thresholds pre-computed for each domain model (pink box), to realise the second main step in MetaCLADE (right white box): overlapping domain hits are selected based on three filtering features. The output of the workflow is an annotation of the ORF, possibly constituted by several domains. The figure illustrates the best expected annotation of a ORF, that is a domain with, possibly, some domain fragments surrounding it. The rectangular pink box illustrates the pre-computed step. For each domain, the CLADE library contains several hundreds of models that are used in MetaCLADE to identify the hits. For domain *D*^1^, considered in the blue cylinder on the left, the model library contains the consensus model (blue coloured line, bottom) and hundreds of CCMs generated from sequences that are spread through the phylogenetic tree of species. Coloured lines represent models constructed from sequences coming from phylogenetic clades coloured on the same colour tone. The blue box on the right illustrates the pre-computation of the domain-specific parameters for the discrimination of positive (light blue, yellow and dark red) from negative (blue, orange and red) sequences. Dots in the plots correspond to sequences. The sequence spaces defined by bit-scores and mean-bit-scores (white plots) and the probability spaces (plots where probabilities are associated to regions) obtained by the naive Bayes classifier are given. **b** Phylogenetic tree of species that generated the CCMs used in MetaCLADE [43]. **c** Histogram reporting the number of CCMs available in MetaCLADE, organised by clades

MetaCLADE workflow is illustrated in Fig. 1a. It takes a dataset of reads (with an average size between 100 and 500 nucleotides) in input and searches for domains using a library of more than two million probabilistic models (CCMs and SCMs) [43] associated to almost 15,000 Pfam domains. For each domain, hundreds of CCMs have been generated from homologous sequences representing the entire phylogenetic tree of life. The spread of species whose sequences were used for CCM construction is illustrated by the phylogenetic tree in Fig. 1b, where each leaf corresponds to a different species. In contrast, the distribution of models constructed from sequences coming from different clades of the phylogenetic tree is illustrated in Fig. 1c. We note that the total number of models constructed from bacterial species is higher than from eukaryotes and even higher than from archaea. Indeed, a species contributes to at most one model for a domain and can be used for constructing several models for different domains. As an example, the number of species considered for Bacteria and Viridiplantae is roughly the same (Fig. 1b) while the number of models constructed from bacterial sequences is an order of magnitude higher than those from Viridiplantae (1.3e + 6 vs 1e + 5, see Fig. 1c).

Given a read, a large number of domain hits is produced from the large number of models. MetaCLADE filters them according to three main criteria, applied one after the other to obtain the most likely annotation for each read. The first selection criteria filters out all overlapping hits for the same domain by using the match best score. Note that for any domain identified in a region of the read, it keeps exactly one domain hit per region. Also, note that a read might contain more than one non-overlapping occurrence of the same domain. This filter constitutes the first rough selection eliminating redundant hits and keeping different domains. The second criteria is the heart of the selection step and filters out most hits by keeping only those having a very high probability of being true hits. Probabilities are estimated in MetaCLADE through a pre-computed step that divides the sequence space of each domain in probability regions describing whether a hit (possibly a fragment of a domain) can be accepted with a certain confidence or not. This filter might eliminate all hits associated to a given domain. Finally, the third step selects hits that might be associated to very similar domains. Often, the hit matches produced by models associated to similar domains are overlapping. MetaCLADE carefully evaluates them and selects the hit with a highest sequence identity to the consensus sequence of the model and the highest bit-score (see “Methods” section).

A main important methodological point in MetaCLADE concerns the explicit estimation of the likelihood of a hit to be a true domain. This estimation is particularly sensitive to the length of the hit, that in MG and MT data might be very small; namely, for each domain, MetaCLADE defines a two-dimensional gathering threshold (GA) by combining bit-score and mean-bit-score of the domain hit and by identifying multiple regions in the two-dimensional sequence space that, with a high probability, contain reliable annotations for short sequences. All computational details of the approach (i.e., algorithms, statistical models and parameter thresholds) are described in the “Methods” section. Differences between MetaCLADE and CLADE are listed in the “Discussion” section.

Annotated MG and MT datasets can be explored to learn about the functional activity of the community. For this, one has to properly evaluate the performance of the methods and this was done on simulated datasets and on several published MG/MT datasets. MetaCLADE’s improvement over HMMer was shown on a simulated dataset, generated from 56 completely sequenced genomes with the addition of sequencing errors, and on five ocean MT samples. Five more environmental samples, produced with 454 GS FLX Titanium technology, demonstrated that MetaCLADE annotation highly improves over InterProScan (run with different libraries). One more environmental sample, produced with Illumina HiSeq 2000 technology, confirmed MetaCLADE’s good performance on datasets of much shorter reads.

To compare MetaCLADE against two state-of-the-art annotation tools designed for short sequences, HMM-GRASPx and UProC, we used environmental samples, a simulated dataset previously employed to evaluate HMM-GRASPx, and two datasets simulated directly from MG sequences. These datasets are different in terms of average read length and species coverage, and they highlight the complementarity of MetaCLADE compared to UProC with respect to reads of shorter (100 bp) and longer (200 bp) size. They help to show that a combination of the two tools can improve performances even more.

### Comparison of MetaCLADE and HMMer on a simulated metagenomic dataset

We simulated a 454-read dataset from 56 fully sequenced genomes—belonging to archaea and bacteria—and accounting for a total size of 187 Mbp (NCBI’s accession numbers and genome sizes are shown in Additional file 1: Table S2.) Genomes have been fragmented with MetaSim [53] and the outcoming clones have been parsed with FlowSim [54], to simulate realistic insertion and deletion errors expected during DNA sequencing. FlowSim was used with the error model of the “454 Titanium FLX” sequencing platform (error rate ∼1*%*). This resulted in about 500,000 reads, with an average size of 523 bp, that were given as input to MetaCLADE.

Even though 454 is becoming nowadays a sequencing platform less and less used, we considered it in order to compare MetaCLADE to HMMer on reads that are not short. Indeed, HMMer was previously shown not to perform well on short fragments [38].

The performance of MetaCLADE and HMMer on the aforementioned ORFs was computed on a ground-truth defined as follows: from each genome, we considered its CoDing Sequence (CDS) in NCBI and retained only those also defined by Swiss-Prot (June 2016 release). CDS regions were further enriched with the available Pfam domain annotation (version 29). Then, the set of true positives has been defined by those ORFs overlapping such Pfam annotations.

Predictions made by MetaCLADE and HMMer have been compared on two levels by considering or not as true positives (or true negatives) the predictions which fell into the same Pfam clan [55] or InterPro family [56]. In the clan-oriented annotation, MetaCLADE and HMMer were able to obtain an F-score of 98.57 and 99.56 respectively. However, MetaCLADE was able to identify more domains (86.2*%*) than HMMer (74.8*%*). Instead, if the comparison does not take into account the clans, the F-scores fall down to 95.22 and 98.16 (with 83.3 and 73.8*%* of recovered domains) for MetaCLADE and HMMer, respectively. In both these analyses, we notice that MetaCLADE presents a higher number of false positives and false negatives with respect to HMMer. This is expected as Swiss-Prot’s annotations—which we used to define the ground-truth—are based on Pfam and hence on HMMer. A synthesis of the comparison is reported in Additional file 1: Table S3.

Finally, it is interesting to point out that the 13.8*%* of MetaCLADE’s missed domains belonged to very small fragments, with an average length of 38 aa. The distribution of *E* values for domains annotated by MetaCLADE is plotted in Additional file 1: Figure S6A (a similar analysis which considers the TrEMBL annotation is available in Additional file 1: Figure S6B).

### Functional annotation of large oceanic metatranscriptomic samples

Domain identification allows to highlight the main functional activities of a community through the identification of the functions supported by the most abundant domains, but also to compare communities and organisms. A more accurate zooming into functional activities hopefully leads to capture missing features of communities’ behaviour.

**Fig. 2:**
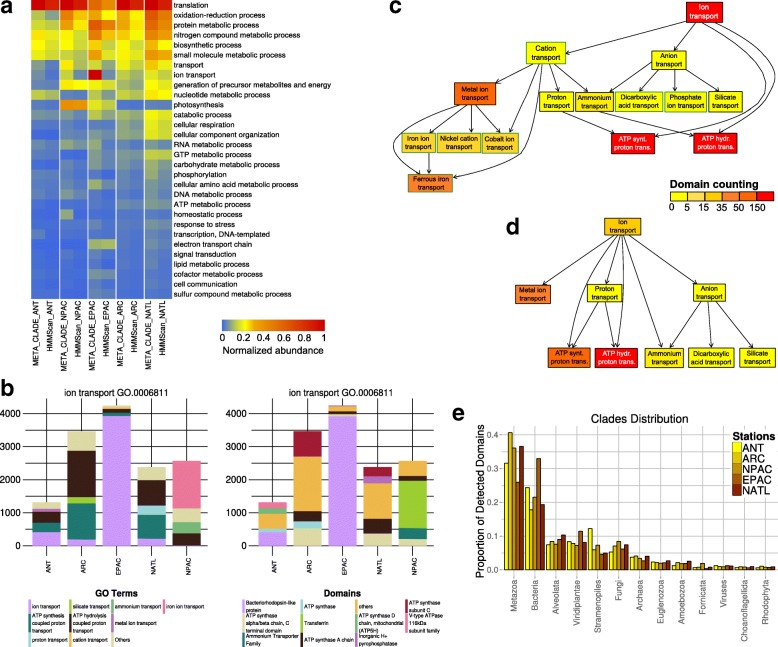
Functional analysis of MT data collected on five ocean sites. **a** Comparative table of domain abundance classification. Rows represent the Metagenomic GO-Slim functional classes that are the most represented, in at least one of the ocean samples. The level of domain abundance (entries of the table; normalised domain abundance, $N^{S}_{I}$ defined in the “Methods” section, is rescaled here in the interval [0,1]) for Antarctic (ANT), North Pacific (NPAC), Equatorial Pacific (EPAC), Arctic (ARC) and North Atlantic (NATL) samples that are described. Samples are arranged in five columns reporting, for each functional class, the level of abundance obtained with MetaCLADE and HMMer. The colour bar ranks high levels of abundance in dark red and low levels in blue. The ranking of Metagenomic GO-Slim functional classes (from top to bottom in the table) is fixed by the average abundance of a domain in the five samples detected by MetaCLADE. Note that only the most abundant subset of GO-Slim classes is reported. **b** MetaCLADE analysis of domains belonging to the GO-term “ion transport” (GO.0006811). Results are displayed by GO-terms association (left) and by domain name (right). Column heights correspond to the estimation $N^{S}_{I}$ of domain abundance relative to each sample *S* and functional class *I* (see the “Domain abundance” section in the “Methods” section). For each environmental sample, the abundance of the first five most represented domains in the sample is plotted (note that each column has five colours.) **c** Hierarchical tree graph of GO-terms for “ion transport” obtained with MetaCLADE and described in **b** for the ANT sample. The count of domains classified with a given GO-term in ANT is represented by the colour of the associated box. The colour scale represents the number of domains identified for a GO-term. Red corresponds to >150 domain hits. Each box in the tree graph is coloured independently of its position in the tree graph because each domain is associated to a single box. There is no cumulative effect in the counting. **d** Hierarchical tree graph of GO-terms for “ion transport” obtained with HMMer. The GO-terms are associated to domains identified by HMMer in the ANT sample, compared to **c**. **e** Distribution of species originating CCMs used to annotate the five MT datasets. Due to the different number of reads in the datasets (and hence, the variable number of identified domains), we report the proportion of species, organised in clades, for each dataset

The functional annotation of domains in the five oceans MT datasets in [57] demonstrates a sharp difference in relative abundance of domains found by MetaCLADE compared to HMMer (hmmscan) (Fig. 2a). MetaCLADE shows that the larger amount of domains it detects falls coherently in functional classes of interest for specific environments, reaching a much better resolution of sign ificant terms among all Metagenomic GO-Slim functional classes. Certain functional classes, such as “translation”, are overrepresented for both MetaCLADE and HMMer, as expected. Others are characteristic of certain environmental conditions, and they are only detected by MetaCLADE. The numerical comparison involving all Metagenomic GO-Slim functional classes (partly visualised by the heat-map in Fig. 2a) is reported in Additional file 1 (see the “Methods” section for the normalisation procedure).

A striking example is the “ion transport” functional class for the EPAC and ANT samples, where HMMer annotation completely misses the large presence of bacteriorhodopsin-like domains in EPAC, as illustrated in Fig. 2a, b. In other environments, such as the ANT sample, there is a much weaker presence of these domains but their existence is nevertheless captured. In particular, MetaCLADE annotation is much finer than HMMer annotation as seen in the complexity of the tree graphs associated to the “ion transport” GO-term in Fig. 2c, d. Note the red colour, representing the highest abundance, given to the node “ion transport” in the MetaCLADE tree graph of Fig. 2c compared to the yellow colour, corresponding to a weaker abundance, in the HMMer tree graph of Fig. 2d. MetaCLADE tree graph is much more detailed and precise in the annotation of domains: it contains six nodes (corresponding to distinct GO-terms) more than the HMMer tree graph. The set of domains for the GO-term “metal ion transport”, for instance, is represented by just one node of 44 identified domains in the HMMer tree graph (Fig. 2d), while it is detailed by a more complex MetaCLADE tree graph of 165 identified domains, associated to iron ion, nickel cation, cobalt ion and ferrous iron transport GO-terms (Fig. 2c). This association to specific functional roles of the identified domains can help biologists to better characterise the metabolic regimes of the sample. Overall, MetaCLADE uniformly annotates more domains and with a more specific functional association than HMMer. The same tree graph analysis was realised for all Metagenomic GO-Slim functional classes, and the functional variability, through which annotated domains span within each tree graph, was estimated by counting the number of nodes (corresponding to distinct GO-terms) in the MetaCLADE and HMMer tree graphs. The values of the analysis are reported in Additional file 2, and they confirm, at large scale, that MetaCLADE annotation provides a more refined functional description than HMMer.

In Fig. 2a, some functional classes appear as the most represented in exactly one environmental sample. This is the case for the pyrophosphates in NATL, the transferrin and the ammonium transporter in NPAC. Others are shared by several samples. They might be present in the remaining samples as well, but relatively less represented (as for the bacteriorhodopsin-like domains in EPAC and ANT discussed above, for instance, illustrated in Fig. 2b). This comparative information is crucial for zooming in the functional activity of an environment.

Finally, one should notice the distribution of species providing the homologous sequences generating CCMs used by MetaCLADE to annotate domains in the five oceanic samples (Fig. 2e). These eukaryotic read sequences were mostly annotated by “eukaryotic” CCMs generated from Metazoa and Alveolata domain sequences. A large contribution from other organisms, such as Bacteria, is also present as expected. We notice a large presence of annotations from “bacterial” CCMs for EPAC. These annotations mostly concern three domains (bacteriorhodopsin-like, *S*-adenosyl-L-homocysteine hydrolase and cyclosome subunit 3—Apc3 domains) covering together the 12% of all EPAC CCM annotations and the 38% of all “bacterial” CCM annotations for EPAC. These are the domains whose “bacterial” CCMs cover alone more than the 1% of all CCM annotations; note that the bacteriorhodopsin-like domain alone covers more than 25% of “bacterial” CCM annotations for EPAC. The list of domains that have been annotated by MetaCLADE with bacterial/eukaryotic/archaea/viral CCMs is given in Additional file 3. See also Additional file 4.

### Identification of divergent domains by conserved small motifs

**Fig. 3:**
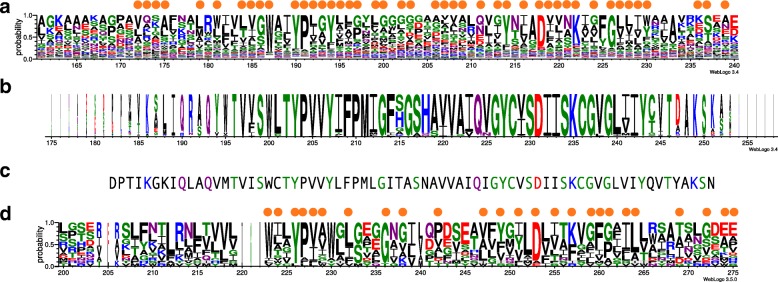
Conserved motif in bacterial rhodopsin sequences annotated by MetaCLADE in the MT dataset EPAC. **a** Conservation profile of the MetaCLADE CCM fragment generated by the *Geodermatophilus obscurus* (strain ATCC 25078/DSM 43160/JCM 3152/G-20; Actinobacteria) sequence of the rhodopsin-like domain used by MetaCLADE to annotate environmental sequences in EPAC [57]. An orange dot is located above all positions in the profile when one of the three top residues with the highest frequency appears as highest frequency residue in the corresponding position of the conservation profile in **b**. **b** Profile generated from the alignment of 371 environmental sequences annotated by MetaCLADE with CCMs of the rhodopsin-like domain and missed by HHMer. The letter height in the logo is proportional to the number of sequences in the alignment that contain the letter at a specific position, and the letter thickness is proportional to the number of gaps in the alignment at that position. **c** Rhodopsin fragment sequence from the dinoflagellate *Prorocentrum donghaiense* found in the NR database and matching, with *E* value 3e −22 and sequence identity 78%, the longest environmental sequence among the 371 annotated by MetaCLADE. Note that the fragment has been aligned to the profile in **b** for a visual inspection of conserved positions. **d** Conservation profile HMM of the Pfam bacterial rhodopsin domain PF01036 (fragment). As in **c**, positions are aligned with the profile in **b** for best visualisation. An orange dot is located above a position in the profile when one of the three top residues with the highest frequency appears as highest frequency residue in the corresponding position of the conservation profile in **b**

MetaCLADE multi-source annotation strategy is used with the purpose of identifying very divergent domain sequences lying in reads. In fact, CCMs are probabilistic models that describe closely specific sequences and they can capture conserved patterns that are specific of homologs niches and that are missed by SCMs. As a consequence, CCMs for a domain have the possibility to describe domain sequences in greater detail and span a greater space of homologous sequences, possibly very divergent. For instance, in Fig. 3a, we consider the conservation profile of the sequence alignment associated to a CCM used in the annotation of the rhodopsin-like domain in MG fragments, missed by HMMer as discussed above, but whose expression is expected in the Equatorial Pacific [58, 59]. With this and other CCMs, MetaCLADE could annotate 371 sequences in EPAC that could not be detected by HMMer in [57] due to the strong sequence divergence (Fig. 3d). The conservation profile of the alignment of the 371 environmental sequences is reported in Fig. 3b. It is very conserved and corresponds to a portion of the rhodopsin-like domain. This conserved pattern makes the third of the length of the entire domain. The rest of the sequence is divergent and remains with no annotation. One can visually appreciate the stronger similarity of the CCM profile (Fig. 3a) to the MG sequences (Fig. 3b) compared to the Pfam SCM profile (Fig. 3d) of the bacterial-like rhodopsin. Indeed, 48 positions of the CCM profile versus 25 of the Pfam SCM profile match the alignment of the MG sequences (that is, given a position, the most represented amino acid in the MG profile is one of the first three best represented amino acids in the CCM/SCM profile at that position).

Note that the motif identified by MetaCLADE in the eukaryotic MG sample was recently identified in the dinoflagellate *Prorocentrum donghaiense* [60] (Fig. 3c) with an alignment comprised by homologs from *Oxyrrhis marina* and bacteria. The conserved positions, characteristic of the dinoflagellate sequence [60], are recovered in the alignment of our MG sequences, confirming MetaCLADE functional annotation.

MetaCLADE demonstrated that its algorithmic strategy allows for the identification of conserved small motifs in MG samples and opens up the possibility of a systematic characterisation of environmental motifs.

### Improved annotation of MG/MT datasets compared to InterProScan

We ran MetaCLADE and InterProScan [61] with five different libraries (Pfam [55, 61, 62], Gene3D [63], TIGRFAM [64], PRINTS [65] and ProSite [66]) on five publicly available MG and MT datasets (listed in Table 1; see Additional file 1). For these datasets, the sequenced reads had been pre-processed with the EBI Metagenomics pipeline [67] leading on which the annotation has been realised.

**Table 1:** # of ORFs annotated by five different domain annotation tools

MG/MT datasets	# Predicted ORFs	Gene3d	Pfam	TIGRFAM	PRINTS&Prosite	MetaCLADE	MetaCLADE +UProC
# of ORFs annotated by each tool							
Puerto Rico Rainforest soil metagenome	520 791	189 138	181 777	15 155	27 710	**260 953**	**266 380**
Arctic Winter marine ecosystem	358 095	113 307	104 098	6 835	15 324	**150 786**	**158 496**
Bone sample from Vindija Neanderthal	74 705	12 793	9 717	612	933	**18 589**	**22 673**
Human gut metagenome	45 770	17 873	18 981	905	2 002	**27 028**	**29 645**
Human gut metatranscriptome	37 209	10 297	9 699	183	714	**16 522**	**20 479**
# of ORFs annotated by one specific tool							
Puerto Rico Rainforest soil metagenome	520 791	16 482	1 090	2 473	138	**37 811**	**43 222**
Arctic Winter marine ecosystem	358 095	11 564	580	1 703	159	**20 531**	**28 227**
Bone sample from Vindija Neanderthal	74 705	1 824	50	294	14	**4 663**	**8 742**
Human gut metagenome	45 770	1 368	55	27	7	**4 477**	**7 093**
Human gut metatranscriptome	37 209	1 172	20	4	7	**3 983**	**7 938**

Number of ORFs (# of ORFs) predicted in reads with FragGeneScan and annotated by different tools. Largest annotations are reported in bold. See Fig. 4 and Additional file 1: Figures S7-S10, S12-S15 for additional information

**Fig. 4:**
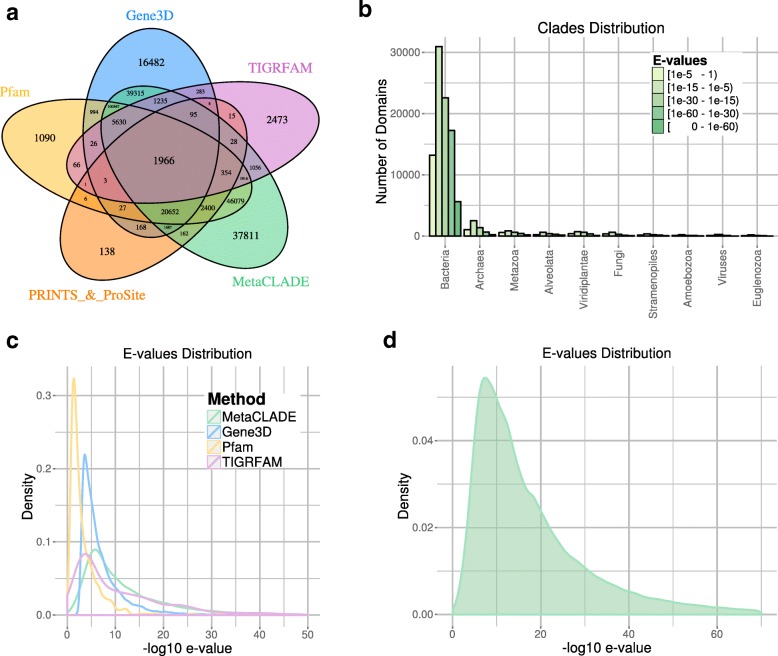
Read annotations of the Puerto Rico Rainforest MG dataset obtained with InterProScan and MetaCLADE. **a** Domain annotation of the five tools: Pfam (yellow), Gene3D (blue), TIGRFAM (purple), PRINTS&ProSite (orange) and MetaCLADE (green). The Venn diagram representing the number of reads annotated by one or several tools is reported. **b** Distribution of species originating CCMs used to annotate the dataset with MetaCLADE. **c** Distributions of *E* values associated to the sets of domains identified in an exclusive manner by each tool. For instance, for MetaCLADE, we considered 37,811 domains (see **a**). *E* values are plotted on the *x*-axis using a − log10 scale. **d** Distribution of *E* values associated to all domains identified by MetaCLADE. As in **c**, *E* values are plotted on the *x*-axis using a − log10 scale

These datasets differ in number of reads and difficulty (measured by the number of annotated domains that could be identified). The performances of InterProScan on the five domain libraries differ greatly (Table 1). The first observation comes from the number of annotated domains shared by the five libraries, which is very reduced, indicating the complementarity of their domain models (see Fig. 4a for the Puerto Rico Rainforest dataset and Additional file 1: Figures S7A-S10A for the other four datasets). One observes that Pfam and Gene3D annotate the largest number of domains, together with MetaCLADE that largely agrees with them. In Fig. 4a, for instance, over 260,000 domains annotated by MetaCLADE, only 38,811 are identified exclusively by MetaCLADE, while all others are found by at least another tool. TIGRFAM and PRINTS&ProSite (the union was considered) annotate the least, and their annotation is largely covered by other libraries. In particular, notice that MetaCLADE annotates a large number of reads that are missed by InterProScan for all five datasets. Table 1 reports the number of ORFs annotated by each library (top) and those exclusively annotated by a single library (bottom). MetaCLADE shows a high number of uniquely annotated ORFs, and, in this respect, it clearly demonstrates to go far beyond InterProScan based on different domain libraries. Moreover, on all datasets, the distribution of *E* values associated to MetaCLADE annotation shows a higher statistical confidence compared to InterProScan with all its libraries. This is illustrated by the density curves in Fig. 4c and Additional file 1: Figures S7C-S10C where the peak of the MetaCLADE curve lies at the rightmost side compared to all other domain annotation tools. In particular, this is true for the Gene3D library, whose peak corresponds to the acceptance threshold for this tool. This allows to explain why MetaCLADE seems to perform poorly on the domains exclusively annotated by Gene3D. In fact, Gene3D uses the *E* value threshold 1e −4 which is for most domains too permissive. Domains were exclusively identified by Gene3D with an average *E* value of 1e −7, while domains exclusively identified by MetaCLADE have an average *E* value of 1e −12.

We note that all the 2473 domains annotated exclusively by TIGRFAM correspond to signatures/domains that are unknown to Pfam (version 27, for which CCMs have been generated) and therefore to MetaCLADE.

### Identification of motifs in short reads: the example of ABC transporters

MetaCLADE is also suitable for technologies producing very short reads like Illumina HiSeq 2000 sequencing system. We analysed the predicted ORFs left without annotation by InterPro from one run of the O’Connor lake dataset. The dataset contains 1,315,435 input reads and 1,211,131 predicted ORFs, with an upper bound on the missed ORFs of 104,304. InterProScan annotated 273,903 ORFs, leaving unannotated 937,228 ORFs. The two sets of predicted and unannotated ORFs have a mean ORF length of 123 bp, with a minimum of 100 bp and a maximum of 135 bp. MetaCLADE analysed the 937,228 unannotated ORFs and succeeded to annotate 57,356 of them. The distribution of *E* values for MetaCLADE annotations is shown in Additional file 1: Figure S11. The list of the most abundant identified domains is given in Additional file 1: Table S6. The domain ranked first is the ABC transporter type 1.

The presence in the MG dataset of annotated sequences presenting some sequence similarity to the known ABC transporters is an indicator of potential metabolic activities that we wish to discover. To support confidence on this identified group of sequences, we scanned them to see if we could find motifs that are known to characterise the ABC transporter domain (https://www.ebi.ac.uk/interpro/entry/IPR000515). For this, we considered all 1109 environmental sequences annotated as ABC transporter type 1 by MetaCLADE (Additional file 1: Table S6) and selected the ones with an *E* value smaller than 1e −4. There are 945 sequences with an average length of 36.9 residues. We ran MEME [68] on them to find the 10 most significant motifs. Among these motifs, we identified the EAA motif, a 20 amino acid-conserved sequence known to occur in ABC transporter type 1 (https://www.ebi.ac.uk/interpro/entry/IPR000515) (See motif 9 in Additional file 1: Table S7; strictly speaking, we found a portion of the known EAA motif, where the submotif EAA −−−−G occurs [69].). The consensus sequences of the 10 motifs (for example, FNLLGDGLRDALDPR for motif 1 and GAILTEAALSFLGLG for motif 9 in Additional file 1: Table S7) were used as query to search the NR database. For all consensus sequences, most of the hits found (> 95%) matched ABC transporters. In rare cases, BLAST [70] retrieved, in addition to the great majority of ABC transporters, other transport systems permeases or hypothetical proteins.

The presence of known motifs favourably supports the finding, and MetaCLADE proves to be able to extract useful functional information even from very short reads. In this respect, Additional file 1: Table S6 shows that MetaCLADE annotations can substantially change the estimations of domain abundance in MG samples compared to estimations realised with HMMer. This confirms what was already observed for the ocean MT datasets, where MetaCLADE allowed for a more precise functional comparison.

### Sensitivity of MetaCLADE on the distribution of species generating the models

**Fig. 5:**
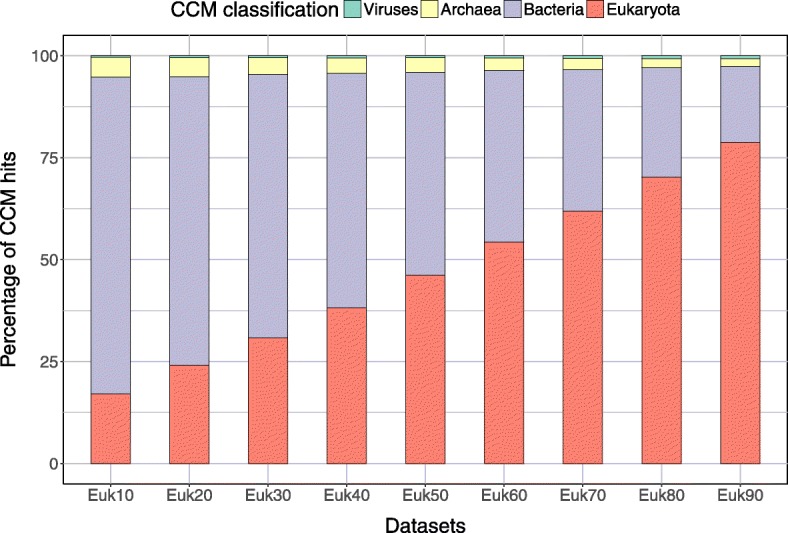
Distributions of species whose sequences generated models for MetaCLADE annotation. Analysis of nine simulated datasets named “Euk*x*” containing *x*% of reads coming from eukaryotic sequences. For increasing values of *x*, one observes a proportionally higher number of CCMs coming from eukaryotes (red) that have been used for the annotation of the dataset. The proportion of bacteria (violet), archaea (yellow) and viruses (green) is reported for each dataset

To analyse MetaCLADE’s sensitivity on the distribution of species from which models have been generated compared to species where reads come from, we verified the distribution of species generating models used for the annotation of nine simulated datasets of reads. These datasets contain short fragments coming from species belonging to bacteria, viruses, archaea and eukaryotes. Specifically, they are constructed by gradually incrementing (by 10%) the number of eukaryotic sequences in them (see the “Methods” section). The nine resulting datasets have been annotated with MetaCLADE, and the origin of the CCMs used is reported in Fig. 5. As expected, in the annotation process, MetaCLADE tends to use models close to the communities represented in the dataset; namely, the number of models generated from eukaryotic sequences used for annotation is proportional to the quantity of eukaryotic reads in the datasets. This observation holds true for real datasets as illustrated in Figs. 2e and 4b (see also Additional file 1: Figures S7B-S10B).

### Comparison with UProC

**Fig. 6:**
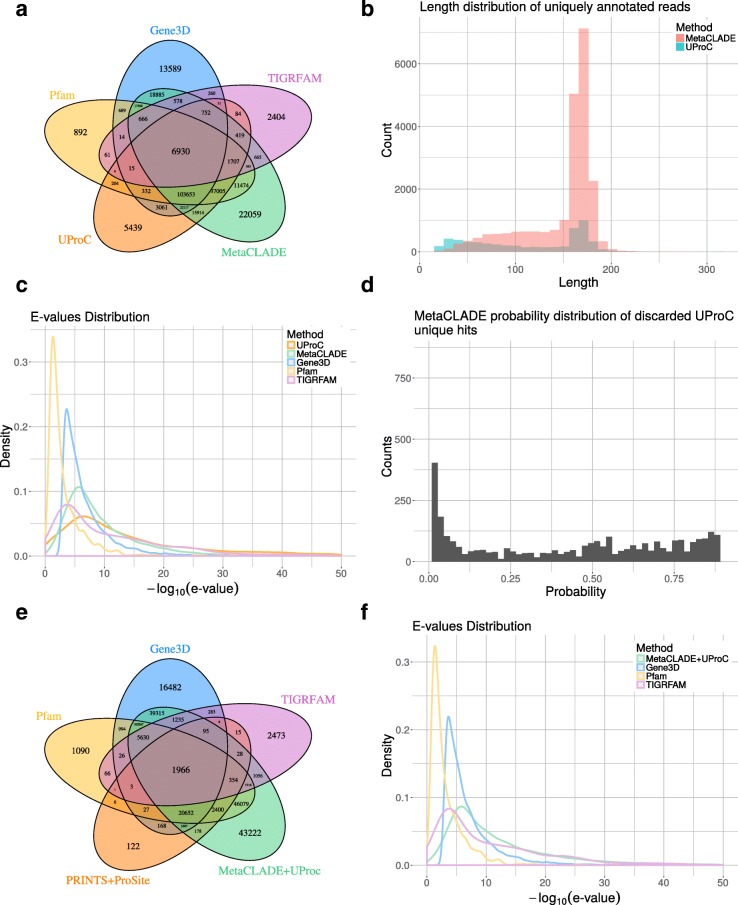
Read annotations of the Puerto Rico Rainforest MG dataset obtained with InterProScan, UProC and MetaCLADE. **a** Domain annotation of five tools: Pfam (yellow), Gene3D (blue), TIGRFAM (purple), UProC (orange) and MetaCLADE (green). The Venn diagram representing the number of reads annotated by one or several tools is reported. **b** Length distribution of reads annotated exclusively by either UProC or MetaCLADE. **c** Distributions of *E* values associated to the sets of domains identified in an exclusive manner by each tool. For instance, for MetaCLADE, we considered 22,059 domains (see **a**). *E* values are plotted on the x-axis using a − log10 scale. **d** Distribution of probabilities associated to those exclusive UProC domain annotations that have been detected by MetaCLADE but discarded because of the probability threshold 0.9. **e** Venn diagram as in **a** of Fig. 4, but where MetaClade is replaced by MetaCLADE+UProC. **f** As in **c**, but where MetaCLADE is replaced by MetaCLADE+UProC

UProC is a very fast protein classification tool designed for large-scale sequence analysis. It is much faster than profile-based methods, like MetaCLADE, and in MG datasets was demonstrated to achieve high sensitivity [38]. We tested whether MetaCLADE, on real datasets, is identifying more domains than UProC or not. The answer depends on the average length of the reads in the dataset. We took the five environmental samples considered in the section “Improved annotation of MG/MT datasets compared to InterProScan” (see Additional file 1: Table S4), ran UProC on them and compared to MetaCLADE results. For the Rainforest MG dataset, the analysis is reported in Fig. 6a. As illustrated in Fig. 6b, the 5439 reads that are uniquely annotated by UProC are either of very small size, <50 aa, or much larger, >150 aa. In contrast, the 22,059 MetaCLADE exclusive annotations do not concern very small reads but rather reads with larger size >50 aa, and particularly >150 aa. The *E* value density distribution curve of UProC annotations (see Fig. 6c and “Methods” section) highlights reasonably low *E* values showing a high confidence in most UProC domain annotations. The second best curve is MetaCLADE’s curve, placed on its left, followed by the InterProScan curves.

A quite large number of reads exclusively predicted by UProC is also predicted by MetaCLADE but not selected by MetaCLADE because of its probability threshold set at 0.9. More precisely, they cover less than 50% of the UProC exclusively predicted reads. By looking at the confidence of these domain predictions, a large number of these predicted reads have very low probability (see Fig. 6d). This observation suggests that, for these domains, the CLADE’s library does not cover properly the spread of evolutionary variability of the domains.

In Additional file 1: Figures S12-S15, we report the analysis of the other four MG and MT datasets. All these datasets confirm that most uniquely annotated UProC reads are very short (<50 aa) compared to reads uniquely annotated by MetaCLADE (see Additional file 1: Figures S12B-S15B). The density curves of the UProC’s associated *E* values occupy the right-hand side of the plots (see Additional file 1: Figures S12C-S15C) with a peak that indicates an optimal average *E* value for UProC reads. Moreover, from the distribution of domain lengths for uniquely annotated reads in Additional file 1: Figure S16, we observe that, depending on the dataset, for a given domain, the read multiplicity can be much larger for MetaCLADE than for UProC. The reason is anchored on the way UProC and MetaCLADE handle sequence information. Indeed, if the space of sequences in the FULL Pfam dataset associated to a domain spans well over diversified homologous sequences, then UProC, which exploits all FULL Pfam sequences, can produce highly confident predictions. If the number of MetaCLADE models is small compared to the number of diversified FULL Pfam sequences, it might represent well only a part of the diversification and fail in predicting homology on the unrepresented pool of sequences. In contrast, if the FULL Pfam dataset does not span well over the entire set of homologous sequences, then MetaCLADE might be able to reach those diverged sequences that cannot be reached by UProC by exploiting its probabilistic models.

### A first comparison with HMM-GRASPx using an assembly-based approach

Assembly algorithms can be used prior to functional annotation of a given MG dataset. We evaluated MetaCLADE’s performance against HMM-GRASPx [37], a state-of-the-art assembly-based annotation method. HMM-GRASPx is characterised by a profile-guided assembly phase in which assembled protein contigs are verified through a HMMER realignment of Pfam profiles. Hence, a domain family is assigned to the reads by mapping them back to verified contigs. In order to provide a fair comparison of the two methods, we decided to annotate the assembly generated by HMM-GRASPx with MetaCLADE and transfer this annotation on the reads by mapping them back to the assembly as done by HMM-GRASPx. Unmapped reads were also separately annotated with MetaCLADE. In parallel, we considered another assembly approach, based on the construction and annotation of a gene catalog [71, 72], where input reads are assembled using a canonical assembly pipeline, ORFs are predicted and putative gene sequences are clustered in order to create a non-redundant set (i.e. the gene catalog). The latter was annotated with MetaCLADE. Reads were finally mapped back to the catalog and the annotation transferred accordingly. Unmapped reads were also separately annotated with MetaCLADE. We compared MetaCLADE based on both assembly approaches against HMM-GRASPx.

To assess annotation performances, the experiment was run on the dataset used in [37] which was augmented in order to better reflect the size of modern metagenomic datasets. More precisely, a set of 20 million paired-end reads was generated from a simulated marine dataset with uneven coverage and read length of 100 bp. A total of 303 Pfam domain families that are involved in some important metabolic pathways were selected as input for HMM-GRASPx and MetaCLADE as in [37] (details are reported in the “Methods” section).

The results of our comparison are reported in Table 2 showing that MetaCLADE always outperforms HMM-GRASPx regardless of the assembly method considered. MetaCLADE, however, performed better considering the gene catalog as a reference. The same observation still holds when clans are evaluated, that is when domain hits of the same clan (with respect to the gold standard) are counted as true positives.

**Table 2:** Comparison of HMM-GRASPx and MetaCLADE against a simulated 100-bp marine data set with uneven coverage

Pathway	HMM-GRASPx						MetaCLADE/HMM-GRASPx-Assembly^c^						MetaCLADE/GC-Assembly^d^					
	TP	FP	FN	Recall	Prec	F-score	TP	FP	FN	Recall	Prec	F-score	TP	FP	FN	Recall	Prec	F-score
“Strict” domain annotation^a^																		
KO00010	159 243	2 216	39 938	79. 9	98.6	88.3	171 168	5 384	24 845	87.3	97.0	91.9	179 972	7 397	14 028	92.8	96.1	*94.4*
KO00020	182 706	11 691	74 704	71.0	94.0	80.9	201 808	24 786	42 507	82.6	89.1	85.7	223 671	20 452	24 978	90.0	91.6	*90.8*
KO00030	101 449	589	17 761	85.1	99.4	91.7	107 065	833	11 901	90.0	99.2	94.4	113 446	859	5 494	95.4	99.2	*97.3*
KO00051	436 432	17 970	192 918	69.3	96.0	80.5	482 994	50 714	113 612	81.0	90.5	85.5	553 728	41 566	52 026	91.4	93.0	*92.2*
KO00620	237 743	11 756	100 903	70.2	95.3	80.8	263 011	27 306	60 085	81.4	90.6	85.8	297 162	21 038	32 202	90.2	93.4	*91.8*
KO00680	595 304	24 101	229 802	72.1	96.1	82.4	633 158	74 709	141 340	81.8	89.4	85.4	695 625	70 569	83 013	89.3	90.8	*90.1*
KO00910	219 501	16 171	86 939	71.6	93.1	81.0	238 683	30 435	53 493	81.7	88.7	85.0	264 438	25 270	32 903	88.9	91.3	*90.1*
KO00920	74 851	138	37 662	66.5	99.8	79.8	82 977	1 098	28 576	74.4	98.7	84.8	105 794	603	6 254	94.4	99.4	*96.9*
“Clan-based” domain annotation^b^																		
KO00010	161 459	0	39 938	80.2	100.0	89.0	176 369	183	24 845	87.7	99.9	93.4	187 308	61	14 028	93.0	100.0	*96.4*
KO00020	194 397	0	74 704	72.2	100.0	83.9	226 585	9	42 507	84.2	100.0	91.4	244 114	9	24 978	90.7	100.0	*95.1*
KO00030	102 038	0	17 761	85.2	100.0	92.0	107 867	31	11 901	90.1	100.0	94.8	114 275	30	5 494	95.4	100.0	*97.6*
KO00051	454 402	0	192 918	70.2	100.0	82.5	533 583	125	113 612	82.4	100.0	90.4	594 999	295	52 026	92.0	100.0	*95.8*
KO00620	249 492	7	100 903	71.2	100.0	83.2	288 167	2 150	60 085	82.7	99.3	90.3	317 646	554	32 202	90.8	99.8	*95.1*
KO00680	618 689	716	229 802	72.9	99.9	84.3	701 356	6 511	141 340	83.2	99.1	90.5	756 885	9 309	83 013	90.1	98.8	*94.3*
KO00910	235 260	412	86 939	73.0	99.8	84.3	267 624	1 494	53 493	83.3	99.4	90.7	288 068	1 640	32 903	89.7	99.4	*94.3*
KO00920	74 989	0	37 662	66.6	100.0	79.9	84 053	22	28 576	74.6	100.0	85.5	106 392	5	6 254	94.4	100.0	*97.1*

^a^Only hits having same Pfam domain with respect to the ground-truth are counted as true positives.

^b^Domain hits that belong to the same clan with respect to the ground-truth are counted as true positives.

^c^Annotation obtained by applying MetaCLADE on the assembled contigs of HMM-GRASPx.

^d^Annotation obtained by applying MetaCLADE on the gene catalog

Largest values are reported in italics

The same comparison had also been carried out on a simulated dataset of about 10 million 200-bp paired-end reads (keeping the same average coverage and composition of the 100-bp read dataset). Here again, MetaCLADE outperformed HMM-GRASPx (Additional file 1: Table S13), especially when considering HMM-GRASPx’s assembly as a reference for MetaCLADE. As a matter of fact, the lower number of reads led to a more fragmented gene catalog and a lower performance of MetaCLADE on these assembled sequences. However, it was not possible to increase the read number (e.g. to 20 million) due to the excessive amount of computational resources (more than 128 GB of RAM) demanded by HMM-GRASPx. In fact, the application of HMM-GRASPx seems limited to datasets of modest size (in terms of both read and profile number).

Overall, even though MetaCLADE achieved very good performances in this assembly-based scenario, we should emphasise that it had been specifically tailored to work with relatively short fragments. Nevertheless, it can definitely benefit from read assembly (possibly in combination with CLADE [43] for the treatment of long sequences). For this reason, we envisage the introduction of a preliminary assembly phase in a future implementation of our tool.

### Comparison with HMM-GRASPx and UProC on datasets of 100- and 200-bp reads

To investigate further MetaCLADE performance with very short reads, characterising the growing number of Illumina sequencing MG datasets available, we generated two sets of sequences, 100 bp (1,226,882 reads) and 200 bp (682,380 reads) long, from the Guerrero Negro Hypersaline Microbial Mats dataset (GNHM; see Additional file 1). GNHM was used in [38] to demonstrate UProC performance on short reads versus profile-based methods and, indeed, in [42], it was shown that profile hidden Markov models substantially lose sensitivity on shorter reads. The two datasets have been evaluated by considering the annotations obtained either with Pfam (version 27) or with CLADE domain library (also based on Pfam27).

Table 3 shows a slightly better performance of UProC compared to HMM-GRASPx and MetaCLADE on GNHM for 100-bp reads with a gold standard set by Pfam annotation. The behaviour becomes less sharp when the gold standard is CLADE, characterised by a larger number of domains. In particular, when clans are considered, MetaCLADE and UProC produce comparable F1-scores, of 57.7 and 58.9 respectively. As 200-bp reads are concerned, MetaCLADE outperforms UProC and HMM-GRASPx regardless of whether clans are considered or not. Notice the high F-scores reached by MetaCLADE, 84.6 and 81.7 versus 74 and 68.9 reached by UProC on the two gold standards when clans are considered (Table 4). It is also interesting to notice that for the 200-bp reads, MetaCLADE identifies a much larger number of true positives and a smaller number of false negatives than UProC, for both gold standards and independently on whether clans are considered or not. In particular, when considering the gold standard set by CLADE and clans, MetaCLADE identifies 80,000 domains more than UProC, and a smaller number of false negatives and false positives.

**Table 3:** Comparison of UProC, HMM-GRASPx and MetaCLADE on the Guerrero Negro Hypersaline microbial Mat project (100-bp reads)

Tool	TP	FP	FN	Recall	Precision	F-score
Pfam gold standard						
UProC	323 659	31 539	357 182	47.5	91.1	*62.5*
MetaCLADE	291 896	57 955	388 945	42.9	83.4	56.6
HMM-GRASPx	325 689	41 155	355 152	47.8	88.8	62.2
MetaCLADE+UProC	370 274	72 132	310 567	54.4	83.7	65.9
UProC+MetaCLADE	378 449	70 063	302 392	55.6	84.4	*67.0*
UProC _clan_	328 910	26 288	351 931	48.3	92.6	63.5
MetaCLADE _clan_	309 629	40 222	371 212	45.5	88.5	60.1
HMM-GRASPx _clan_	334 480	32 364	346 361	49.1	91.2	*63.9*
MetaCLADE+UProC _clan_	389 454	52 952	291 387	57.2	88.0	*69.3*
UProC+MetaCLADE _clan_	390 388	58 124	290 453	57.3	87.0	69.1
CLADE gold standard						
UProC	308 483	48 077	476 068	39.3	86.5	*54.1*
MetaCLADE	286 458	48 696	498 093	36.5	85.5	51.2
HMM-GRASPx	296 742	69 211	487 809	37.8	81.1	51.6
MetaCLADE+UProC	361 794	64 085	422 757	46.1	85.0	59.8
UProC+MetaCLADE	366 221	66 114	418 330	46.7	84.7	*60.2*
UProC _clan_	336 302	20 258	448 249	42.9	94.3	*58.9*
MetaCLADE _clan_	323 009	12 145	461 542	41.2	96.4	57.7
HMM-GRASPx _clan_	328 729	37 224	455 822	41.9	89.8	57.1
MetaCLADE+UProC _clan_	405 734	20 145	378 817	51.7	95.3	*67.0*
UProC+MetaCLADE _clan_	406 370	25 965	378 181	51.8	94.0	66.8

The first table considers Pfam27 annotations as gold standard, while the second one uses CLADE27. Each table is made of two sub-tables where we evaluate annotation on exact domains (top) and on clans (bottom). Annotations with domains of the same clan are counted as true positives

Largest values are reported in italics

**Table 4:** Comparison of UProC, HMM-GRASPx and MetaCLADE on the Guerrero Negro Hypersaline microbial Mat project (200-bp reads)

Tool	TP	FP	FN	Recall	Precision	F-score
Pfam gold standard						
UProC	252 245	28 101	161 239	61.0	90.0	72.7
MetaCLADE	307 711	50 380	105 773	74.4	85.9	*79.8*
HMM-GRASPx	288 152	38 152	125 332	69.7	88.3	77.9
MetaCLADE+UProC	319 829	55 189	93 655	77.3	85.3	81.1
UProC+MetaCLADE	326 133	56 556	87 351	78.9	85.2	*81.9*
UProC _clan_	256 847	23 499	156 637	62.1	91.6	74.0
MetaCLADE _clan_	326 217	31 874	87 267	78.9	91.1	*84.6*
HMM-GRASPx _clan_	293 267	33 037	120 217	70.9	89.9	79.3
MetaCLADE+UProC _clan_	338 609	36 409	74 875	81.9	90.3	*85.9*
UProC+MetaCLADE _clan_	339 285	43 404	74 199	82.1	88.7	85.2
CLADE gold standard						
UProC	240 762	43 085	244 393	49.6	84.8	62.6
MetaCLADE	302 583	63 491	182 572	62.4	82.7	*71.1*
HMM-GRASPx	262 511	64 833	222 644	54.1	80.2	64.6
MetaCLADE+UProC	316 656	68 490	168 499	65.3	82.2	72.8
UProC+MetaCLADE	322 760	70 325	162 395	66.5	82.1	*73.5*
UProC _clan_	264 787	19 060	220 368	54.6	93.3	68.9
MetaCLADE _clan_	347 936	18 138	137 219	71.7	95.0	*81.7*
HMM-GRASPx _clan_	290 155	37 189	195 000	59.8	88.6	71.4
MetaCLADE+UProC _clan_	363 667	21 479	121 488	75.0	94.4	*83.6*
UProC+MetaCLADE _*clan*_	364 641	28 444	120 514	75.2	92.8	83.0

The first table considers Pfam27 annotations as gold standard, while the second one uses CLADE27. Each table is made of two sub-tables where we evaluate annotation on exact domains (top) and on clans (bottom). Annotations with domains of the same clan are counted as true positives

Largest values are reported in italics

The two plots in Additional file 1: Figure S17 display the relation between precision and recall, in order to evaluate the performance of the two tools on the two datasets, when the gold standard is CLADE and clans are considered. MetaCLADE displays a slightly better behaviour than UProC for fixed recall values. Notice that for very small recall values and, hence, very high hit scores, UProC detects a higher number of false positives (yet quite small) compared to MetaCLADE. This is seen with the behaviour of the curves in the zoomed plots in Additional file 1: Figure S17, bottom. Precision-recall curves for UProC and MetaCLADE with Pfam-based gold standard and clan-based annotation are shown in Additional file 1: Figure S18.

### A measure of the improvement obtained by combining MetaCLADE and UProC

By combining MetaCLADE and UProC, we obtain an improved quality of the functional assignments of sequences of GNHM on both the 100-bp and the 200-bp datasets. Indeed, for the two datasets, we considered the annotation realised by MetaCLADE augmented with UProC annotation on those reads left unannotated by MetaCLADE, and called this approach MetaCLADE+UProC (see the “Methods” section). Vice versa, we considered the annotation realised by UProC augmented with MetaCLADE annotation on those reads left unannotated by UProC, and called this approach UProC+MetaCLADE (see the “Methods” section). On both GNHM datasets, for the 100- and 200-bp reads, UProC+MetaCLADE outperformed on exact domain annotation and MetaCLADE+UProC on clan annotation. This is expected because UProC, based on word matching, is intuitively similar to very conserved CCMs that are very close to known sequences. Most importantly, the performance of their combination is bringing a clear improvement in terms of number of correctly predicted domains independently on the gold standard and on the dataset (Tables 3 and 4). In Additional file 1: Figure S17, the precision-recall curve for MetaCLADE+UProC shows that the addition of unique UProC annotations to MetaCLADE increases the number of false positives and therefore decreases precisions, as also seen in Table 3. The advantage in using a combined approach like MetaCLADE+UProC relies on the increase of the recall (since false negatives decrease) counterbalanced by a small decrease in precision (since false positives slightly increase).

The combination MetaCLADE+UProC has been tested also on the Rainforest MG dataset, where it produced a much larger number of read predictions as illustrated in Fig. 6e, accompanied by a distribution of *E* values showing high confidence (see Fig. 6f and compare it to Fig. 4a, c). MetaCLADE+UProC behaviour on the other four MG/MT samples in Table 1 is reported in Additional file 1: Figures S12E-S15E and shows a high improvement in performance associated to high confidence *E* values (Additional file 1: Figures S12F-S15F).

## Discussion

MetaCLADE was especially designed to consider the partial information contained in domain fragments, localised in reads. For this, we defined a powerful two-dimensional domain-dependent gathering threshold and we use multiple models to represent each domain, possibly characterising small conserved motifs for the domain. In future development, we foresee to improve the tool in several ways (see also [43]):

**More domains and new models for an improved MetaCLADE annotation.** New CCMs could be added to the library with the hope to reach novel and unrepresented evolutionary solutions for a domain. An obvious improvement could be obtained by extending the library with the set of new domains included in Gene3D and TIGRFAM. The motifs represented in PRINTS and ProSite could be also considered and the associated profiles handled in MetaCLADE. Note that MetaCLADE package provides the program to pre-compute gathering thresholds for all domain models. This allows the user to compute appropriate thresholds based on new CCMs.

**Constructing a library of conserved small motifs.** The search for sequence motifs in an environmental sample might be realised with a computationally costly “all against all” read comparison. Alternatively, starting from the most conserved patterns comprised in CCMs, we can generate a repertoire of significant motifs specific of each domain in order to improve hit selection criteria. A systematic classification of these motifs might lead to datasets of motifs that could be used as environmental signatures of metabolic activities.

These “environmental patterns” could be also used to find new domains in environmental samples with MetaCLADE. The advantage in this search approach, compared to an “all against all” strategy, is that patterns are constructed starting from domains, possibly functionally annotated and that this annotation could be used to associate a potential functional role to new domains discovered through the pattern.

**Annotation of longer sequences.** Availability of long reads and read assembly in contigs allow reconstructing longer stretches, and possibly entire, ORF sequences. In this case, one could replace the third filter in MetaCLADE with DAMA [73], to reconstruct the best domain architecture as done in CLADE.

**Reduction of the number of redundant models in MetaCLADE.** Some of the probabilistic models in MetaCLADE library are expected to be redundant, and a suitable handling of these models, after clustering, should help to increase the speed of the method and to preserve the same predictive power. Future development of MetaCLADE will reduce the number of redundant models representing domains.

**New criteria to filter overlapping hits in MetaCLADE.** Different domain hits could be selected by exploiting further the characteristics of the two-dimensional space of sequences pre-computed for the domains. For instance, one could privilege the domain hits with larger bit-score/mean-bit-score distance from the closest negative in the space. These filtering conditions could improve the annotation and need to be tested at large scale.

**MetaCLADE differences with CLADE.** MetaCLADE has been designed with the purpose of annotating MG and MT reads. It exploits the multi-source annotation strategy introduced in CLADE and the CLADE model library, but it handles the models and their output in a different manner. Indeed, the CLADE pipeline combines the output of its rich database of probabilistic models with a machine-learning strategy in order to determine a set of best predictions for each domain sequence. Then, DAMA [73] is used to find the best domain architecture, by using information on domain co-occurrence and by exploiting multi-objective optimisation criteria.

Neither CLADE machine-learning algorithm nor DAMA are used in MetaCLADE. In fact, the characteristics of MG and MT reads, compared to full protein sequences, are their short lengths and the presence of multiple sequencing errors in them compared to full-length ORFs. Hence, they demand the design of a special computational protocol taking into account the particular nature of the data; namely:

1. CCMs cannot be used with tailored GA thresholds as in CLADE. Instead, we introduce an original bi-dimensional gathering threshold that is specifically designed for evaluating short hits. For each domain, we compute a probability space on which to evaluate hits. This is done with a naïve Bayes classifier. Note that the computation of such a probability space depends on an appropriate generation of positive and negative sequences on which evaluate models for a domain.
2. CLADE machine-learning algorithm cannot be used for protein fragment identification. Indeed, CLADE works well with the full domain annotation of known genomes. In its design, it explicitly considers *E* value, hit length, consensus on multiple domain hits and clade-specific hits. On the other hand, read annotation should be less sensitive to sequence errors and hit length and should disregard the species the sequence comes from. In MetaCLADE, we do not use a SVM combining the above characteristics but instead we create a simpler pipeline of hit selection.
3. DAMA, the tool used in CLADE to reconstruct protein architectures, cannot be used on short reads. Indeed, reads might be long enough to contain at most one adjacent pair of domains and certainly cannot provide information to evaluate the contextual annotation of a domain within a potential domain architecture. In MetaCLADE, knowledge of adjacent pairs of domains could be considered but we left it for future developments.

## Conclusion

MG and MT datasets have been explored mostly to learn about which and in what abundance species are present in the community. Learning about the functional activity of the community and its subcommunities is a crucial step to understand species interactions and large-scale environmental impact. Ecological questions, such as how limited availability of abiotic factors in an ocean shape most abundant genes in a community, or how temperature affects eukaryotic phytoplankton growth strategies, for example, can be approached with an accurate domain annotation and a precise functional mapping. In this respect, one might need to zoom into functional activities and metabolic pathways employed by the environmental communities that might involve non-highly expressed genes. This means searching for lowly abundant domains that, through cooperation, might imply important functional effects. In order to capture common and rare entities in a given environment, functional annotation methods need to be as precise as possible in identifying remote homology.

Nowadays, the bottleneck resides in the annotation step, directly influencing an appropriate quantitative estimation of the domains. Here, we show how MetaCLADE, based on a multi-source annotation strategy especially designed for MG/MT data, allows for the discovery of patterns in very divergent sequences and provides a way to overcome this fundamental barrier. With the ongoing generation of new MG/MT data, unknown sequences will augment in number and probabilistic models are expected to play a major role in the annotation of sequences that span unrepresented sequence spaces. This point is clearly shown in our comparison with UProC, which is based on k-mer recognition, and therefore particularly adapted to the identification of already known domain sequences. By construction, UProC approach cannot be successful on unknown diverged domain sequences, a context where probabilistic domain modelling fully reveals its predictive power.

## Methods

This section explains MetaCLADE’s methodology and the datasets used in the analyses. The differences between MetaCLADE and CLADE [43] are presented in the “Discussion” section. The time complexity is explained in Additional file 1.

The testing datasets were designed to fit current technological characteristics. We considered that Illumina represents nowadays the dominant technology in most sequencing projects. Currently, the HiSeq and MiSeq platforms are able to produce pair-end reads of 150 bp and 300 bp, respectively (with a much higher throughput for the first one). Such fragments in practice might be even shorter after the required low-quality-base trimming. For these reasons, we considered testing datasets of reads of increasing lengths. More precisely, we chose 100 bp and 200 bp as read lengths. However, we also chose to test MetaCLADE on simulated 454 fragments (mean length 523 bp) in order to prove its versatility and to show the annotation improvement as read length increases. This test is particularly important in view of the efforts from current technologies to increase read length.

### The multi-source annotation strategy and the CLADE library

Widely used search methods based on sequence-profile and profile-profile comparison, such as PSI-BLAST [45], HMMer [47] and HHblits [49], are based on a mono-source annotation strategy, where a single probabilistic model, generated from the consensus of a set of homologous sequences, is used to represent a protein domain. The mono-source strategy typically performs well when sequences are highly conserved. In this case, the consensus model captures the most conserved features in domain sequences and it can be successfully used to find new domains in databases of sequences, sharing the same features as the original sequence. However, when sequences have highly diverged, consensus signals become too weak to generate a useful probabilistic representation and models constructed by global consensus do not characterise domain features properly.

To overcome this fundamental bottleneck, CLADE [43], a domain annotation tool tailored to full genomes, introduced a multi-source annotation strategy, where several probabilistic models are used to represent a protein domain. For each Pfam domain *D*^*i*^, CLADE considers the FULL set of homologous sequences *S*^*i*^ in Pfam [61] associated to *D*^*i*^, and for some representative sequences *s*_*j*_ in *S*^*i*^ (see below), it constructs a model by retrieving with PSI-BLAST [45] a set of sequences similar to *s*_*j*_ from the NCBI NR database. The probabilistic model generated in this way displays features that are characteristic of the sequence *s*_*j*_ and that might be very different for other sequences *s*_*k*_ in *S*^*i*^. The more divergent the homologous domain sequences *s*_*j*_ and *s*_*k*_ are, the more models constructed from these sequences are expected to display different features. It is therefore important for a domain *D*^*i*^ to be represented by several models that can characterise its different pathways of evolution within different clades. These probabilistic models are called clade-centered models (CCMs). The multi-source annotation strategy has proven more efficient than the mono-source annotation strategy when applied to full genomes [43]. In particular, due to their closeness to actual protein sequences, CLADE’s CCMs are shown to be more specific and functionally predictive than the broadly used consensus models.

MetaCLADE is based on the multi-source annotation strategy and employs the CLADE library that includes the Pfam sequence consensus models (SCM) and at most 350 clade-centered models (CCM), with an average of 161 models per domain. The representative sequences associated to these models are selected in order to span most of the tree of life, the underlying idea being that evolutionary patterns can be found in species that are very far apart in the tree. This amounts to more than 2.5 millions probabilistic models.

### The MetaCLADE’s pipeline

MetaCLADE’s pipeline is illustrated in Fig. 1. It is based on two main steps, dedicated to the identification of domain hits and on their selection, and on a pre-computed learning step setting domain-specific two-dimensional thresholds used in domain selection.

#### Identification of domain hits

MetaCLADE takes as input a set of MG/MT sequences to be annotated and the CLADE model library. More specifically, the input sequences coming from a dataset of reads are expected to be (subsequences of) open reading frames (ORFs). Alternatively, one can use MetaCLADE on the six reading frame translations of the reads.

Each sequence is scanned with the model library in order to identify all domain hits. Each hit is defined by a bit-score, that is the PSI-BLAST/HMMer score associated to the match, and by a mean-bit-score, that is the bit-score of the hit divided by its length. These two scores are used to evaluate the likelihood of the hit to represent a true annotation (see the “Selection of domain hits” section; for the computation of the likelihood see the “Pre-computed two-dimensional gathering thresholds for domain identification” section).

The output of this first step of MetaCLADE is a set of hits, each one defined by a domain family *D*, a probabilistic model *M* associated to *D*, a bit-score and a mean-bit-score.

#### Selection of domain hits

The second step of the pipeline filters the set of hits as follows:

1. All pairs of overlapping hits associated to the *same* domain (i.e. the overlap region covers at least 85% of both hit lengths) are processed with the intention of eliminating their redundancy. Therefore, for each overlapping pair, we retain only the best hit (i.e. with the higher bit score). The filtering is realised independently for CCMs and for SCMs.
2. Based on the probability obtained with the naive Bayes classifier [74] applied to each Pfam domain (see the “A naive Bayes classifier sets two-dimensional thresholds for fragmented domains” section), MetaCLADE retains only those hits whose bit-score is greater than a domain-specific lower bound identified by the classifier and whose probability *p* of being a true positive is greater than 0.9. More precisely, such a lower bound is defined as the smallest bit-score of the negative sequences used by the classifier during its training (see the “Pre-computed two-dimensional gathering thresholds for domain identification” section).
3. Hits are filtered according to a *ranking* function based on the bit-score and the identity percentage computed with respect to the model consensus sequence. Specifically, we associate to each hit a real number in the interval [0,1] representing the statistical significance of the bit-score. Such a value is then multiplied by the identity percentage of the hit in order to define the *ranking score*. Therefore, domain hits are ordered by decreasing values of their ranking scores and iteratively discarded if they share at least 10 residues with some domain with a higher scoring hit. Eventually, this allows us to provide a small architecture (usually involving up to two domains, due to read length) for each annotated sequence.

Note that in the third point, the ranking score combines the (statistical significance of the) bit-score and the percentage identity of the match in a product and that these two values are highly correlated. Indeed, one expects higher bit-scores to be associated to higher sequence identities. This means that when two matches differ strongly on their bit-scores, the respective products will not be affected by the percentage identity of the matches. On the contrary, it is when the bit-scores are close to each other that the percentage identity of the matches will play a role by favouring matches with higher sequence identity. Intuitively, MetaCLADE prioritises bit-scores while letting percentage identity play a discriminative role between very close bit-score values.

The output of this filtering step is the ORF annotation with non-overlapping domain hits. Due to the short length of the reads, one expects at most two domains per read, possibly flanked by domain fragments on the right and/or the left. Consequently and in contrast to CLADE, there is no reconstruction of the best architecture with DAMA.

Also, note that the first filter is used to reduce the size of the set of domain hits, possibly huge at the beginning due to redundant predictions of the high number of models. The second filter is used to identify hits having a high probability to be true hits, and it constitutes the core of the filtering process. The third filter is used to identify the best solution, among the ones with highest bit-score, based on motif conservation.

As a consequence of the construction of the probability space for a domain, the second filter asks for domain hits to have a bit-score greater than the smallest bit-score of the negative sequences in the space. This is because negative sequences considered by the classifier are a selected sampling of the space of negatives (see the “Generation of negative sequences” section below); namely, among all negatives generated by the algorithmic procedure, we selected those that lie further away from the origin and that, in consequence, have the highest statistical significance. These selected negative sequences tend to group together further from the origin of the space and to lie at the borderline of regions characterised by positive sequences. Hence, one should properly evaluate the acceptance threshold against this specificity.

### Pre-computed two-dimensional gathering thresholds for domain identification

MG and MT samples demand to annotate domain fragments, possibly of small length. In order to explicitly distinguish small hits from long ones, MetaCLADE directly estimates the likelihood for a small hit to be a positive sequence by considering the bit-score of the hit and also its mean-bit-score; namely, it defines a two-dimensional gathering threshold (GA) for each domain by combining bit-score and mean-bit-score and by identifying multiple regions in the two-dimensional sequence space that, with a high probability, provide reliable annotations for short sequences. Probabilities are estimated with a naive Bayes classifier [74], and the statistical procedure is explained below.

#### Construction of positive and negative training sets

For each domain, MetaCLADE estimates bit-score and mean-bit-score domain-sensitive thresholds. More precisely, it constructs a sequence space for each domain, by defining a set of positive sequences (i.e. actual fragments of the domain) and by generating a set of negative sequences (i.e. sequences wrongly annotated with the domain). Ideally, for each domain, one would like to have a training set comprised of a comparable number of positive and negative sequences.

**Definition of positive sequences.** The training set of positive sequences was constructed as follows. For each domain *D*^*i*^ and for each sequence in the Pfam SEED set of homologous sequences for *D*^*i*^, we created a set of prefixes and suffixes of the sequence to simulate small domain portions coming from the beginning or the end of the domain sequence that may be found in MG reads. The maximum size *M* of prefixes and suffixes was set to 30% of the entire domain sequence length and to a maximum of 100 aa. Hence, fragments were determined by increasing lengths *n*·*L*, where *L* is a constant depending on domain size and *n*=1,2,3…*N* is a multiplicative factor where *N* corresponds to the smallest integer such that *M*≤*N*·*L*. For domains of length between 15 and 75 aa, the constant *L* was set at 5 aa, for sizes >75 aa it was set to 10 aa, and for sizes <15 aa it was set to 1 aa. For large domains, >270 aa (this corresponds to one standard deviation away from the mean in the distribution of domain model sizes as reported in Additional file 1: Figure S1), we expect that reads may lie somewhere in the middle of the domain and therefore we extracted random sequences from the original sequence that were not already covered by small fragmentations of the extremes. Fragment positions were set by randomly choosing their first position along the middle part of the sequence, and fragment lengths were randomly picked from a normal distribution with mean 50 and standard deviation 25. The number of fragments corresponds to ten.

**Generation of negative sequences.** In order to define a set of negative sequences for each model (CCM or SCM) associated to a domain, we generate a large amount of decoy sequences and select as negatives those where the original domain is identified by the model (with an *E* value <1 for CCMs and a positive bit-score for SCMs).

The algorithm generates first sequences with two different methods:

1. A random shuffling of the 2-mers of each SEED sequence
2. The reversal of SEED sequences and checks whether they are negatives or not. If the number of negatives reaches at least the 50% of the positive sequences, then the algorithm stops the search. Otherwise, new sequences are generated with a third method:
3. By constructing a Markov model of order 3 for each domain and by using it to generate random sequences with positional probabilities

Note that in 3, the space of 160,000 (20^4^) 4-tuples is evaluated by assigning a probability to appear in a domain sequence to each 4-tuple. This is done with a pseudo-count, by considering each 4-tuple to appear at least once and by counting the number of occurrences *n* of the 4-tuple in the SEED sequences of the domain. The probability of a 4-tuple is set to $\frac {n+1}{160~000+ N}$, where *N* is the total number of 4-tuple occurrences in the SEED sequences. The Markov model of order 3 is defined on these probability estimations.

Only generated sequences whose original domain has been correctly identified by PSI-BLAST (for CCMs) with an *E* value <1 or by HMMer (for SCMs) with a positive bit-score are considered as negative sequences for the MetaCLADE models (CCMs or SCM, respectively) and are included in their training sets. The usage of different threshold for the two tools, PSI-BLAST and HMMer, is due to the observation that it is easier to produce negatives with PSI-BLAST than with HMMer; therefore, an *E* value threshold <1, much more selective than a positive bit-score, would reduce the large number of accepted negatives for CCMs. The statistical significance and the impact of these thresholds on the space of positive and negative sequences is discussed below (see the “A naive Bayes classifier sets two-dimensional thresholds for fragmented domains” section).

The algorithm estimates the number of decoy sequences that should be generated to obtain the 50% of negative sequences and stops when this estimated number of sequences is generated. For example, supposing there are 100 positive sequences for a domain, then we seek to generate at least 50 negative sequences. If random reshuffling and reversal generate only 10 negative sequences, a Markov model is expected to generate 40 sequences. Since most decoys generated by the Markov model will not be selected as negatives, one estimates the number of decoys that should be generated by the Markov model to obtain 40 negatives and stops the algorithm after such number. The estimation has been realised based on the observation that false positives are found after a very large number of decoy generations: roughly one expects to obtain 1–10 false positives out of 10,000 decoys for SCMs and out of 1000 decoys for CCMs. CCMs lie very close to actual sequences, and for this reason, we expect them to be much more effective in recognising a domain in a random sequence generated by a Markov model of that domain than a SCM. If a domain contains only a few sequences in its SEED set, *n* would be too small to produce a significant bias in the 4-mer probability emission. Therefore, *n* is multiplied by a factor *W* with initial value at 10 and incremented by one until we reach the generation rate of 1–10 negative sequences out of 10,000 decoys for the domain and its SCM. (To estimate the weight, we only use the SCM and no CCMs.) This leads to emission probabilities $\frac {(n+1) \times W}{160,000 + N\times W}$. Note that each decoy is tested against both SCM and CCMs associated to the domain and that it is considered as negative if at least one model identifies the domain in it. If too many negative sequences were produced, then only those that are the most distant from the origin of the sequence space (that is the euclidean distance of the point, defined by the bit-score and the mean-bit-score of a hit, from the origin of the two-dimensional space) are retained, limiting their amount to the number of positive sequences. Note that sequences that are most distant from the origin are those with higher statistical significance. The distribution of sequences generated with Markov models is reported in Additional file 1: Figure S2. They make a total of 39,241,830,000 sequences. In contrast, the first two methods generated a total of 22,816,657.

Positive and negative sequences are described by a bit-score and a mean-bit-score. To avoid an over-representation of sequences with the same bit-score and mean-bit-score, we consider only one representative per fixed values of bit-score and mean-bit-score. This makes the two sets of positive and negative sequences, all domain confounded, to be numerically comparable. The total amount of non-redundant (in terms of their bit-score and mean-bit-score) positive matches is 13,697,142 for SCMs and 7,548,890 for CCMs while the total amount of negatives is 7,569,171 and 6,342,944, for SCMs and CCMs respectively (Additional file 1: Figure S3).

Globally, we ensure the full training set is comprised of roughly 50% of positive sequences and 50% of negative ones (Additional file 1: Figure S4B). This proportion varies from domain to domain and depends on the difficulty to generate correctly annotated random sequences. In Additional file 1: Figure S4, we report the proportions of negative sequences for CCMs and SCMs generated by the first two methods (Additional file 1: Figure S4A) and compare them to the distributions of sequences generated by all three methods (Additional file 1: Figure S4B). Clearly, the third method contributes to the largest number of negative sequences for each domain and establishes the expected numerical balance between the two training sets; all domains confounded.

#### A naive Bayes classifier sets two-dimensional thresholds for fragmented domains

Positive and negative sequences are put together and analysed to obtain best separation parameters for CCMs and SCMs; namely, we use a discrete version of the naive Bayes classifier [74] (downloadable from http://www.cs.waikato.ac.nz/ml/weka/citing.html) to construct learning models for each Pfam domain. The discrete version of the naive Bayes classifier provides a finite partition of the sequence space and an estimation of the probability for a sequence to be a positive or a negative hit. Notice that we realise two different analyses, one on CCMs (generated by PSI-BLAST) and the other on SCMs (pHMMs generated by HMMer), because we cannot immediately compare their bit-scores. By so doing, we determine two distinct separation spaces and appropriate parameters for the two model predictions. In particular, only one probability space is estimated for all CCMs of a domain. All positive and negative sequences, generated for all CCMs, are considered in the same sequence space and the associated probability space is computed.

**Fig. 7:**
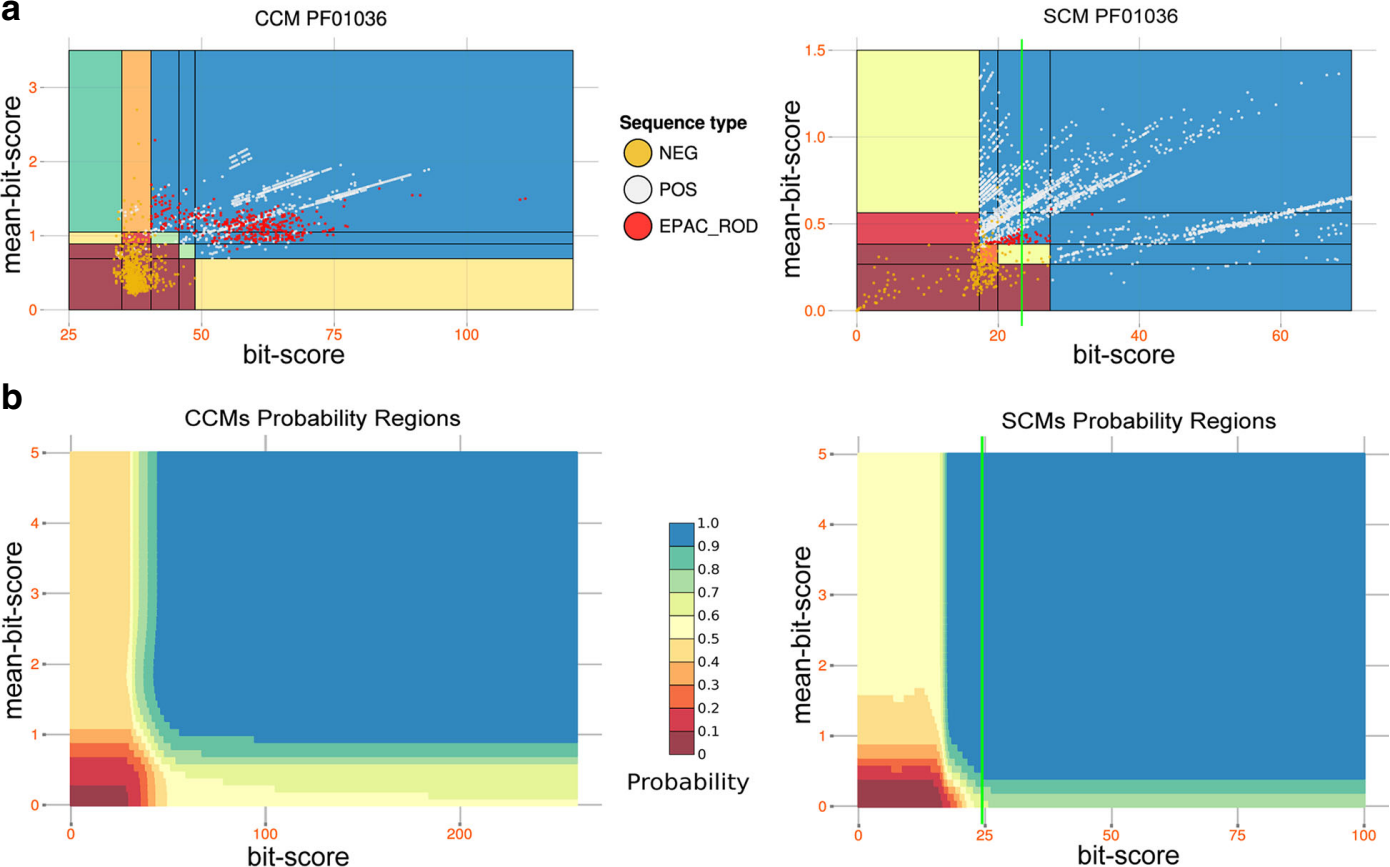
Naive Bayes classifier analysis of Pfam domains. **a** Naive Bayes classifier analysis of the training set evaluating thresholds for positive (cyan dots) and negative (orange dots) hits discrimination of the Pfam domain “bacteriorhodopsin-like protein” (PF01036). The space of positive hits is coloured blue, and the space of negative hits is the complementary one, coloured from green to dark red (see colour scaling). From dark red to blue, each coloured area is characterised by a different probability for a sequence to be a positive sequence. The space of solutions for all CCMs confounded (left) and the SCM (pHMM; right) are described. The red dots correspond to the MG sequences identified by the models in the Equatorial Pacific (EPAC) MT dataset discussed below [57]. The green vertical line on the SCM plot (right) corresponds to hmmscan GA threshold (= 24, for this domain). Note that the grid is discrete and that rectangular regions are coloured with respect to probability intervals. This means that two adjacent regions with the same colour might have associated two different probabilities. **b** The probability space of CCMs and SCMs generated for MetaCLADE; all domains included. Each point of the plot is the average of the corresponding points in all spaces of solutions computed for CCMs and SCMs, respectively. Examples of such spaces of solutions are reported in **a** for the Pfam domain PF01036. The colour scale is defined with respect to probability values associated to the regions. The green vertical line in the SCM plot (right) corresponds to the average hmmscan GA threshold; all domain confounded. Note that if we take the bit-score only as a threshold in our database, we obtain that an average bit-score at 25 in **a** is statistically meaningful, independently of the mean-bit-score. This value is used as a default threshold in http://www.ebi.ac.uk/Tools/hmmer/search/phmmer

Figure 7a illustrates an example of separation of the spaces of positive and negative hits for the CCMs and SCM of a Pfam domain (PF01036), analysed with the naive Bayes classifier. Note that short fragments have small bit-scores with a possibly large mean-bit-score and that negative sequences are characterised by small bit-scores and small mean-bit-scores. Also, the identification of fragmented coding regions (especially important for the annotation of MG/MT datasets, where ORFs that are present in MG/MT reads are fragmented) will only be realised through very low bit-scores because only parts of domains are present. These sequences can be seen in the blue region of positive hits in Fig. 7a, where successively larger fragments appear associated to successively larger scores. They are represented by trails of points in the figure, where small fragments have small scores.

A comparison between the two spaces illustrated in Fig. 7a shows a basic difference between CCM and SCM models. First, notice the much smaller number of negative sequences obtained for the SCM space compared to the CCM space (see “Construction of positive and negative training sets” section). In the CCM space, negative sequences are more clearly separated by both bit-scores and mean-bit-scores from positive ones than in the SCM space. In fact, since CCMs are “closer” to sequences than SCMs, one expects their scores to be higher for positive sequences in CCMs than in SCMs. Also, the usage of a two-dimensional sequence space, determined by bit-scores and mean-bit-scores, improves the separation of positive and negative sequences in MetaCLADE compared to HMMer (hmmscan). In the plot describing the SCM space (Fig. 7a, right), the Pfam GA threshold excludes most of the Bacteriorhodopsin-like protein sequences detected by CCMs. More generally, for all Pfam domains, we computed the difference between the GA threshold associated to the SCM and the mean of the bit-scores for the five best negative sequences identified by the SCM. The distribution of the differences, displayed in Additional file 1: Figure S5, shows a small standard deviation suggesting that MetaCLADE and Pfam/HMMer estimation of the (one dimensional) cutoff is similar. This control shows that naive Bayes classification produces reasonable thresholds when projected in one dimension.

Figure 7b illustrates the general behaviour of the probability spaces for all CCMs and SCMs, all domains confounded. It shows the coherence of the spaces across models of the same domain and highlights bit-scores and mean bit-scores intervals defining rejecting and accepting regions. One observes large regions associated to high probability values (>0.9) accepting true positives.

### Comparison to InterProScan

To compare MetaCLADE to InterProScan, we used several tools and domain model libraries: Pfam [61], TIGRFAM [64], Gene3D [63] and PRINTS & ProSite [65, 66]. Annotations produced by these tools were downloaded from available annotation files provided by the EBI metagenomics pipeline http://www.ebi.ac.uk/metagenomics/pipelines/ (versions 1.0 or 2.0 depending on the dataset). The EBI metagenomics pipeline uses InterProScan (v5.0 for pipeline 1.0 and 5.9-50 for pipeline 2.0) as tool to annotate predicted ORF (using FragGeneScan 1.15) using the domain libraries above. HMMer v3.0 was downloaded from http://hmmer.org and run with default parameters and curated inclusion thresholds (option –cut_ga).

### Comparison to UProC and a combination of UProC and MetaCLADE

UProC [38] (version 1.2.0, downloaded at https://github.com/gobics/UProC) is a tool designed for large-scale sequence analysis. Given a read dataset, UProC decides whether a domain is present or not in a sequence, but it does not point out precisely where the domain is localised in the sequence by defining starting and ending positions, nor it associates an *E* value to the matching. The complementary performance of UProC compared to MetaCLADE suggests to combine the two tools in order to achieve best performance in terms of number of correctly predicted domains. For this reason, we define “MetaCLADE+UProC” by adding to MetaCLADE’s annotations all those obtained by UProC on reads left unannotated by MetaCLADE. In a similar way, we define the symmetric “UProC+MetaCLADE” annotation.

In order to evaluate UProC, we looked for the position of the domain in a read and associated an *E* value to it as follows. For each read annotated with a domain *D* by UProC, we used BLAST (with default parameters) to map it against all Pfam FULL sequences representing *D* and we selected the best hit, if it existed. The *E* value associated to this hit was considered as the UProC *E* value score.

### Comparison to HMM-GRASPx

MetaCLADE was compared to HMM-GRASPx version 0.0.1 [37] (downloaded at http://sourceforge.net/projects/hmm-graspx); other successive beta versions have been tested, but they did not provide better results. HMM-GRASPx is a profile-based method, like MetaCLADE. It considers profile HMMs of protein families as references and uses them to guide the assembly of complete or near-complete genes and protein sequences related to a particular family. The annotation is realised after the assembly with a mapping of the reads on validated contigs.

### The use of clans and InterPro families

In order to evaluate MetaCLADE performance on datasets of simulated sequences, we identify domains at the Pfam clan [55] or InterPro family [56] level. This is done because sequence similarity within domains in the same Pfam clan is usually high and genome annotation is often mislead by domains belonging to the same clan. This is even more true in MG datasets, where one often needs to annotate fragments of a domain displaying a weaker signal due to the reduced length.

Pfam clans are groups of proteins for which common ancestry can be inferred by similarity of sequence, structure or profile HMM [55]. The list of Pfam clans was retrieved at http://ftp://ftp.ebi.ac.uk/pub/databases/Pfam/releases/Pfam27.0/Pfam-A.clans.tsv.gz.

InterPro families represent groups of evolutionarily related proteins that share common functions. Such entries tend to be near full length and typically do not undergo recombination, in contrast to domains [56]. The list of InterPro families was retrieved at https://www.ebi.ac.uk/interpro/download.html.

### Domain abundance

The functional analysis of a MG/MT sample is realised by characterising domain abundance within a functional class with a normalised value between 0 and 1. This normalisation is done by dividing the number of domains detected in a functional class by the total number of domains belonging to the most represented class in the environmental sample. We speak about “normalised abundance”.

A second kind of normalisation is realised with respect to multiple environments, and it is used for comparing domain abundance within the same functional class across these environments. A normalised domain abundance $N^{S}_{I}$, where *S* is the sample and *I* is the domain, is computed as the product of the actual domain abundance per megabase by the average size of all samples. By multiplying by the average size of all samples, we provide an indication of the expected number of domains if all environments had the same size and can compare environments with respect to such estimations.

### Functional analysis of annotated real datasets

In order to validate MetaCLADE on all real MT and MG datasets, we associated a function based on GO classification to both the domains identified with MetaCLADE and the domains identified with HMMer (hmmscan). We used Pfam2GO [75] and annotated biological process terms of GO-Slim [56, 76]. Pfam2GO was retrieved from http://geneontology.org/external2go/pfam2go, and GO-Slim classification for MG was retrieved from http://geneontology.org/page/download-ontology. To highlight the differences between MetaCLADE and HMMer, we compared domain abundance in all GO-term classes. For this, we normalised domain count in each MT dataset with respect to the size of the sample as described above.

### Motif validation

To validate motifs identified by MetaCLADE on the O’Connor lake dataset, we run MEME [68] with default parameters on the MEME Suite 4.11.2 server at http://meme-suite.org/tools/meme.

### MG/MT datasets used in the analyses

#### A dataset of simulated reads generated from bacterial and archaeal genomes

We generated a set of fragmented sequences from a set of 11 archaeal and 44 bacterial fully sequenced genomes. The list of species, NCBI accession numbers and genome lengths is reported in Additional file 1: Table S2. We assumed all species be equally abundant.

In order to generate a set of 500,000 clones, we first used MetaSim [53], according to a normal distribution with a mean of 800 bp and a standard deviation of 100 bp. Then, we applied FlowSim [77] to the set of clones to obtain actual reads simulated with realistic insertion and deletion errors expected during DNA sequencing. More precisely, the simulation was performed according to the FlowSim platform 454 GS-FLX Titanium (error rate ∼1*%*). The read dataset, which contained sequences of ∼523 bp on average, was finally processed with FragGeneScan [78] in order to predict the ORFs. This resulted in about 500,000 reads that were given as input to MetaCLADE.

In parallel, we used information available in the XML file associated to each genome in the EBI site (http://www.ebi.ac.uk/genomes/) to identify coding sequences included in the SwissProt database. For these coding sequences, we retrieved annotated domains and their positions from the InterPro site through the UniProtKB proteins file (http://ftp.ebi.ac.uk/pub/databases/interpro/protein2ipr.dat.gz). Domain annotation of coding sequences was used to evaluate MetaCLADE performance. For this simulated dataset, we selected positive domain hits with a probability threshold of 0.85 estimated by the naive Bayes classifier.

The dataset is available at http://www.lcqb.upmc.fr/metaclade/.

#### Three datasets of simulated reads to compare with UProC and HMM-GRASPx

MetaCLADE was compared to HMM-GRASPx and UProC on the Guerrero Negro Hypersaline Microbial Mats (GNHM) MG dataset [79] used in [38] to demonstrate UProC performance versus profile-based methods on short reads. GNHM was downloaded from http://uproc.gobics.de. GNHM is a dataset presenting a large variety of species of low abundance, with archaeal and eukaryotic species much less abundant than bacterial ones. From GNHM data, following the protocol in [38], we generated two sets of short reads simulating different read lengths of 100 bp and 200 bp, respectively. The only difference with the described protocol was in the latest HMMer, version 3.1b2 was used instead of the previous one (3.0) that was used in [38]. For the two sets of reads, we considered two annotations as gold standard, one realised with HMMer (hmmscan with GA cutoff) and the other with CLADE [43]. Both annotations are based on domains known in Pfam 27.

The comparison between MetaCLADE and HMM-GRASPx was realised on two simulated datasets of reads of length 100 bp and 200 bp, generated from a marine MG dataset containing 23 marine microbial genomes from the *Alteromonas*, *Candidatus*, *Erythrobacter*, *Flavobacteriales*, *Nitrosococcus*, *Photobacterium*, *Prochlorococcus*, *Roseobacter*, *Shewanella*, *Synechococcus* and *Vibrio* groups. Relative abundances of these bacteria were simulated according to their environmental composition [37] with an average coverage of ∼ 27.5X (Additional file 1: Table S11). We considered these 23 bacterial genomes along with a selected number of important metabolic pathways (see Additional file 1: Table S12). Only those Pfam (version 27) domain families involved in each one of these pathways were finally considered (family-pathway association was performed in [37] using KEGG’s release 73.0 of January 2015). The same domain families were taken into account in HMM-GRASPx and MetaCLADE. Illumina-like paired-end nucleotide reads were generated with wgsim (version 0.3.1-r13 obtained from http://github.com/lh3/wgsim, with error rate parameter -e 0.01) and translated into short peptide reads using FragGeneScan [78] (version 1.30, with parameters -complete=0 -train=illumina_10). For each pathway, the gold standard was defined by searching the corresponding Pfam models against the complete proteomes and transferring the annotation to those reads that mapped to a domain hit for at least the 60% of their length. Finally, gene catalogs were built using MOCAT2’s pipeline [30] with default parameters.

The datasets are available at http://www.lcqb.upmc.fr/metaclade/.

#### Nine simulated datasets to test MetaCLADE’s sensitivity to sequence origin

In order to test how MetaCLADE is sensitive to the distribution of species in a dataset, we created nine simulated datasets with increasing percentages of eukaryotic sequences, and we annotated them in order to examine the origin of the CCMs used by MetaCLADE. Specifically, each dataset is composed by 75,000 randomly chosen UniProt coding sequences that had not been picked to build any model of CLADE’s library. The percentage of eukaryotic CDS among the datasets varies from 10 to 90% with steps of 10%. From such CDS, a set of 50 aa fragments has been uniformly extracted in order to reach a 1.5X coverage. Additional file 1: Table S9 describes the simulated datasets (e.g. average CDS length, number of generated fragments). Overall, we employed 3128 bacterial, 9403 eukariotic, 218 archaeal and 2127 viral species and we annotated with 1,397,356 bacterial, 858,908 eukaryotic, 101,171 archaeal and 31,799 viral models (see Additional file 1: Table S10).

#### Real metagenomics and metatranscriptomic datasets

In order to validate MetaCLADE on real data, we analysed eleven MG and MT samples. The characteristics of these eleven datasets, such as number of reads, average read size and sequencing technique used to generate the dataset, whether it is a MG or a MT dataset, are provided in Additional file 1: Table S3. Available websites for download are given in Additional file 1: Table S4.

Five MT samples come from different geographic locations in the oceans, Antarctic (ANT), North Pacific (NPAC), Equatorial Pacific (EPAC), Arctic (ARC) and North Atlantic (NATL) [57]. We have identified ORFs on the reads by analysing six reading frames and annotated them with HMMer and MetaCLADE. These datasets are available upon request addressed to the authors of [57].

Four published MG datasets come from very different environments: soil, ocean, ancient bones and guts. For the gut environment, we also considered a MT sample. These five sets of reads were previously analysed for ORF identification by EBI with FragGeneScan [78]. ORF sequences have been annotated by EBI based on five different domain databases found in InterPro [56]: Pfam [61], TIGRFAM [64], Gene3D [63] and PRINTS & ProSite [65, 66]. The search was realised with InterProScan [80] as the final step of the EBI MG pipeline (http://www.ebi.ac.uk/metagenomics/about). These tools are accessible from http://www.ebi.ac.uk/services/proteins. Comparison with UProC was realised on these five sets.

The O’Connor lake MG dataset (ERP009498) was downloaded from the EBI Metagenomics portal (https://www.ebi.ac.uk/metagenomics/projects/ERP009498). It was realised with Illumina HiSeq 2000 technology and contains 1,315,435 very short reads (123 nt average length). ORFs were identified by EBI.

MetaCLADE positive domain hits are selected with a probability threshold of 0.9.

## Time complexity

MetaCLADE was run on a High Performance Computing architecture. In all analyses reported here, parallel computation is exploited in MetaCLADE first main step and in the first two sub-steps of the second main step. Notice that MetaCLADE can be run on a desktop computer, especially when restricting the analysis to a small subset of domains. However, when considering the full domain library on a large-size dataset of sequences, MetaCLADE can take a large amount of time as highlighted in Additional file 1: Table S7 for all MG/MT samples considered in the article. For further details, see Additional file 1.

## Evaluation measures

In order to evaluate the annotation tools, we used the standard measures of precision (also named positive predictive value, PPV), accounting for how many annotations are correct and defined as $\frac {TP}{TP+FP}$, and recall (also named sensitivity or true positive rate, TPR), accounting for how many correct annotations are selected and defined as $\frac {TP}{TP+FN}$, where *TP* indicates the number of domains that have been correctly annotated, *FN* indicates the number of domains which are in the gold standard but were not found by the tool and *FP* indicates the number of domains that have been wrongly annotated (because they do not appear in the gold standard). The F-score is the harmonic mean of precision and recall, defined as $2\cdot \frac {\text {precision} \: \cdot \: \text {recall}}{\text {precision} \: + \: \text {recall}}$ ($= \frac {2TP}{2TP+FP+FN}$).

In order to plot the precision-recall curves (Additional file 1: Figure S16), we consider those subsets of reads that are annotated by the tool at a fixed threshold. The curve is constructed by varying a threshold that in MetaCLADE and MetaCLADE+UProC runs over *E* values and in UProC runs over UProC scores.

### MetaCLADE software

The pipeline is implemented in Python 2.7 and is available at http://www.lcqb.upmc.fr/metaclade or at https://sourcesup.renater.fr/projects/metaclade/ under the CeCILL Free Software Licence. This includes the annotation tool (MetaCLADE two main steps in Fig. 1) and the program pre-computing domain-specific gathering thresholds (MetaCLADE pre-computed step in Fig. 1). The CLADE model library used in MetaCLADE was constructed based on Pfam database v27 and was released with CLADE [43]. It can be found at http://www.lcqb.upmc.fr/CLADE.

## Additional files

Additional file 1 Additional Figures and Additional Tables (PDF 3796 kb)

Additional file 2 Normalised abundance for all GO-Slim terms/data used to build figure 2A (XLS 74 kb)

Additional file 3 Total number of nodes in the tree-graph rooted on a specific GO-term (XLS 35 kb)

Additional file 4 Percentage of domain hits wrt all domain annotations (% tot) and wrt bacterial CCM annotations (% bacteria) (XLS 127 kb)
