# Supplementary material for: A multi-source domain annotation pipeline for quantitative metagenomic and metatranscriptomic functional profiling
Source: Microbiome. 2018 Aug 28;6:149. doi: 10.1186/s40168-018-0532-2 (PMC6114274; doi:10.1186/s40168-018-0532-2)
Supplement: Supplementary file 1 — Additional Figures and Additional Tables (PDF 3796 kb) [file 40168_2018_532_MOESM1_ESM.pdf]

Supplementary Material

A multi-source domain annotation pipeline for quantitative metagenomic and metatranscriptomic functional profiling

A. Ugarte, R. Vicedomini, J. Bernardes, A. Carbone

1 Time complexity

MetaCLADE runs on High Performance Computing. Parallel computation is exploited in MetaCLADE first main step and in the first two sub-steps of the second main step. For each MG/MT sample, MetaCLADE run time is reported in **Supplemental Table S7**. For example, for the largest dataset analysed in this publication, the Puerto Rico Rainforest dataset, we used 3 354.35 hours to accomplish domain hit identification (MetaCLADE step 1). On 64 cores, this step takes approximately 52 hours. This step is the most time consuming and it is dependent of the time complexity of the search tools HMMer and PSI-BLAST. Domain filtering (MetaCLADE step 2) is faster. The first filter took 183.24 hours, while the second took 48.76 hours. The third and final step in domain selection cannot run in parallel but took only 19.66 minutes. On 64 cores, domain selection (the entire step 2) can be realised in approximately 3.94 hours.

The pre-computed step (the Naive Bayes training step) was realised in a total of 67 637 135 hours, considering a single core. In reality, each probabilistic model analysis was run in parallel on several cores. It is important to notice that this step was executed once, for all datasets analysed in this article. Note that the training spaces constructed in this step can be improved by the addition of new negative sequences to existing ones.

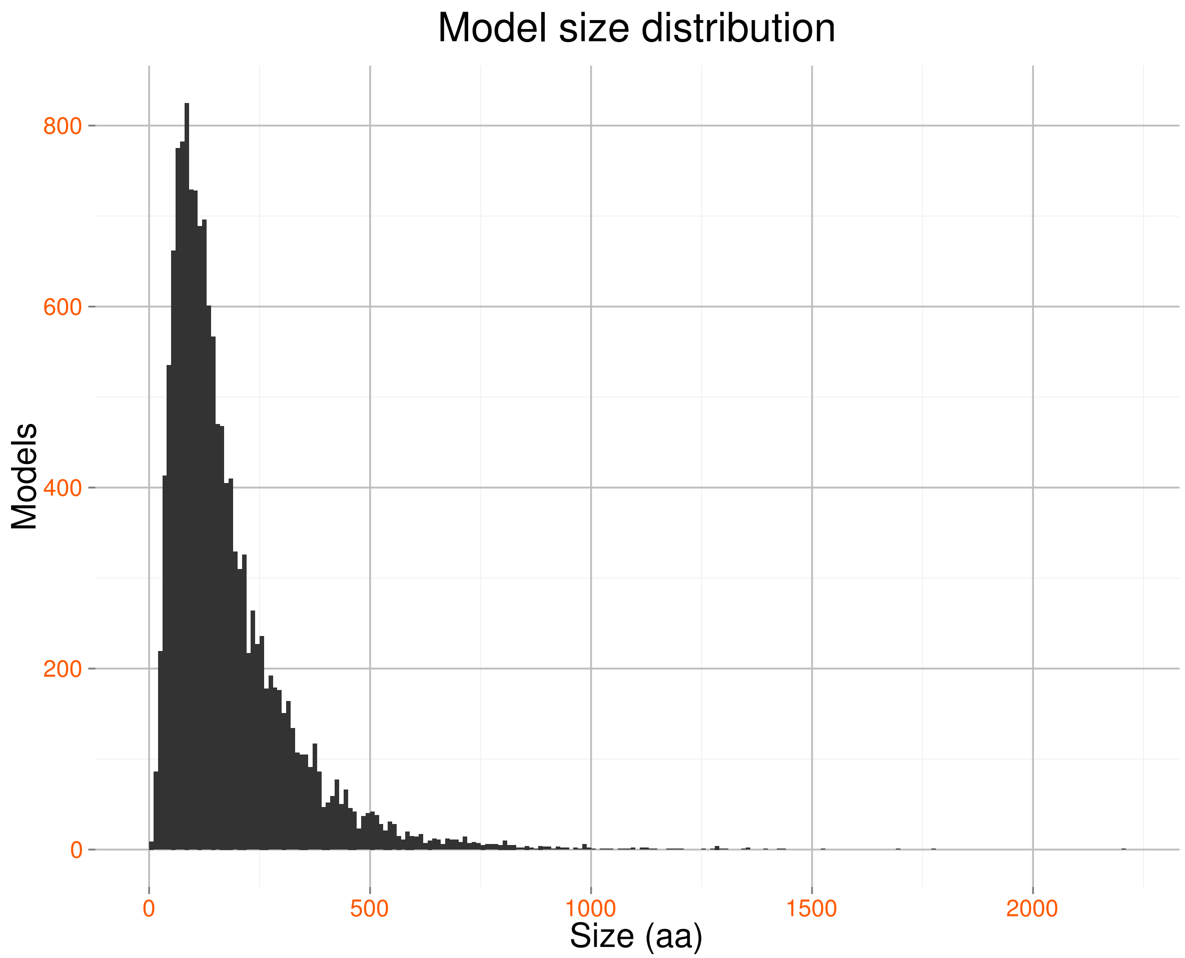

Figure S1: **Distribution of Pfam domain model sizes.** Distribution of the size of the SCMs associated to Pfam domains used in metaCLADE.

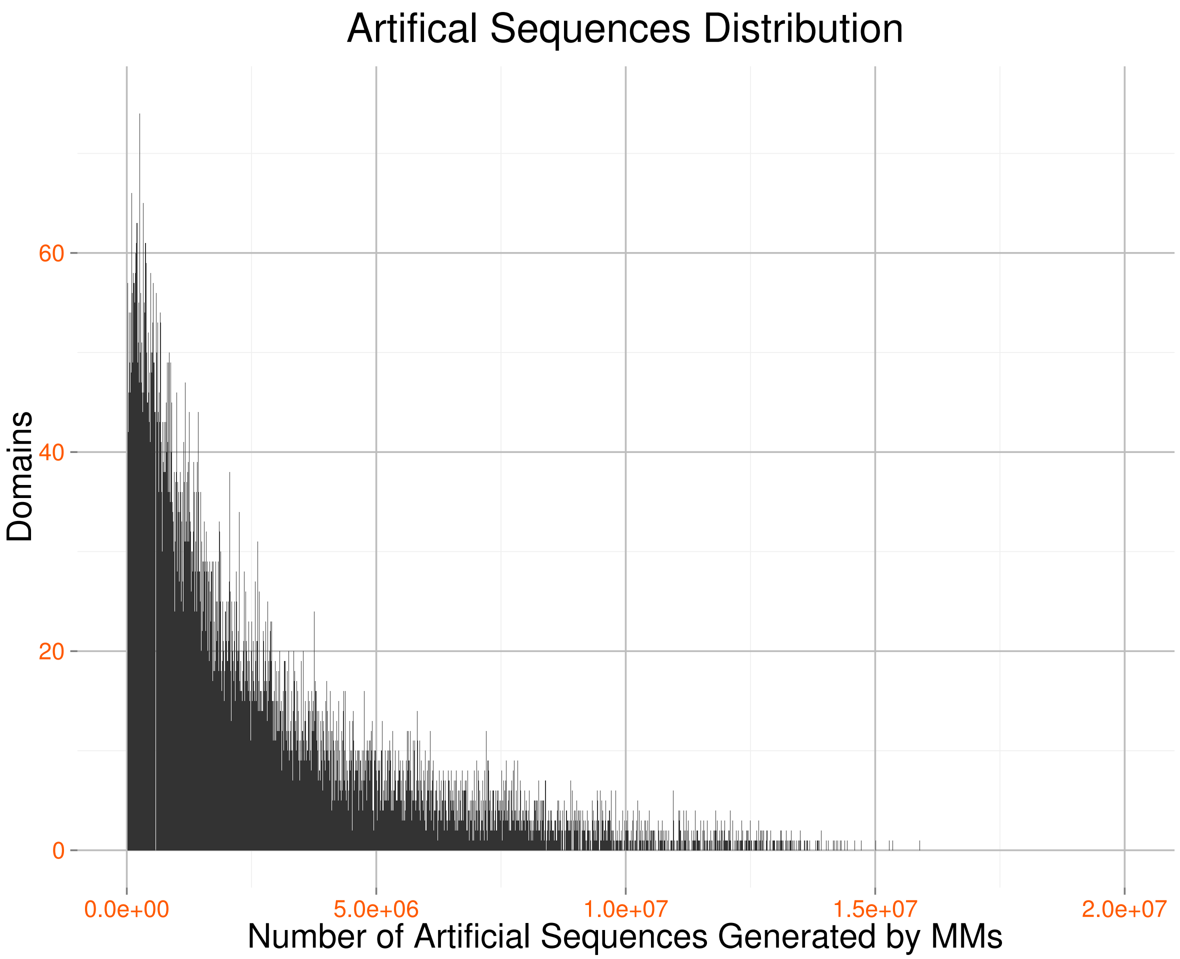

Figure S2: **Distribution of potential negative sequences, generated with Markov models of order 3.** Distribution of all potential negative sequences, all domain models confounded, generated with Markov models of order 3. They make a total of 39 241 830 000 sequences. Note that the identification of these sequences was possible after the generation of about 16 million sequences by Markov models, for each one of the 14 831 domains in Pfam.

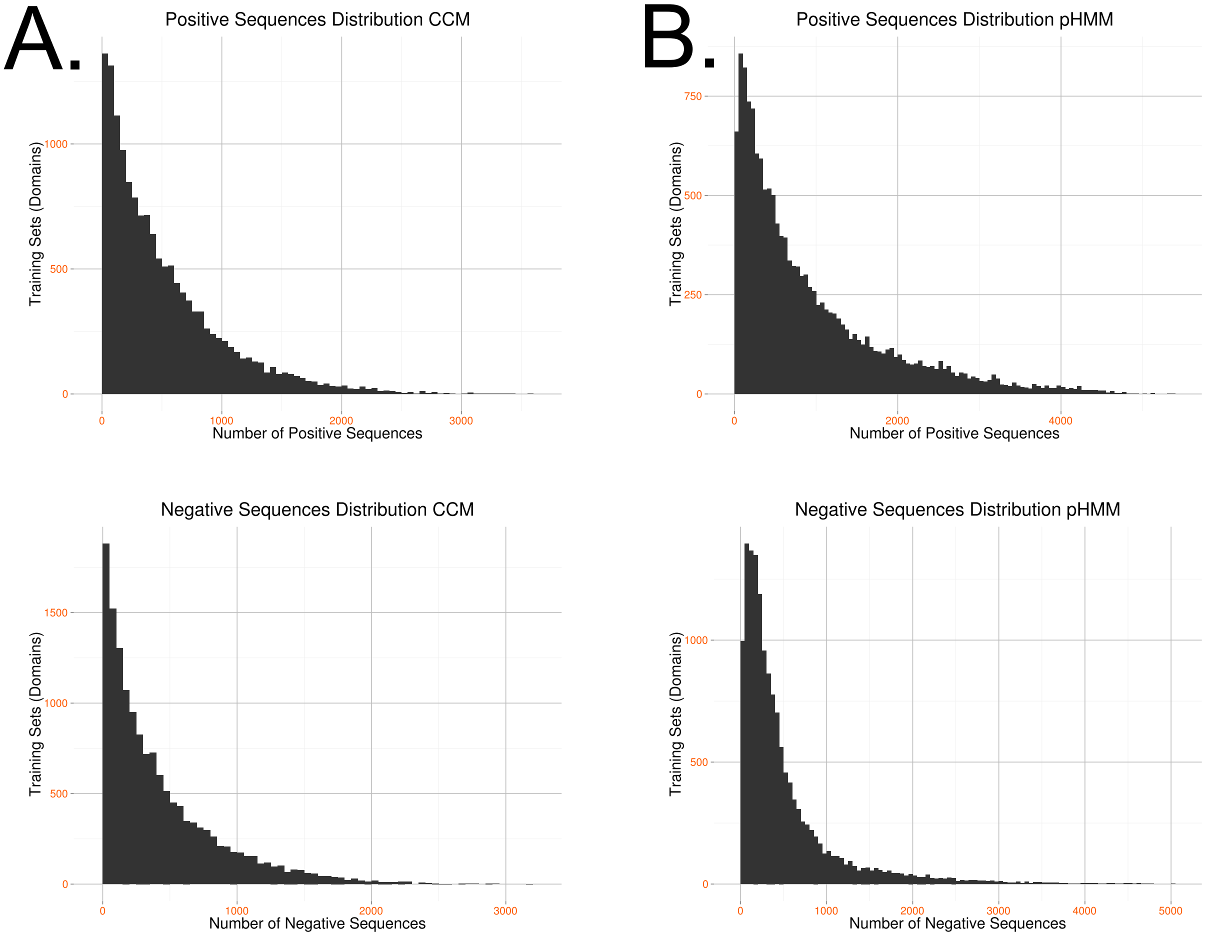

Figure S3: **Positive and negative sequences distributions for CCMs and SCMs (pHMMs), all domains confounded.** Distribution of positive and negative sequences in the training sets associated to CCMs (**A**) and SCMs (pHMM; **B**), all domain confounded. For CCMs, there are about 7 millions generated positive sequences and 6 millions negative ones. For SCMs (pHMM), there are about 13 millions generated positive sequences and about 7 millions negative ones.

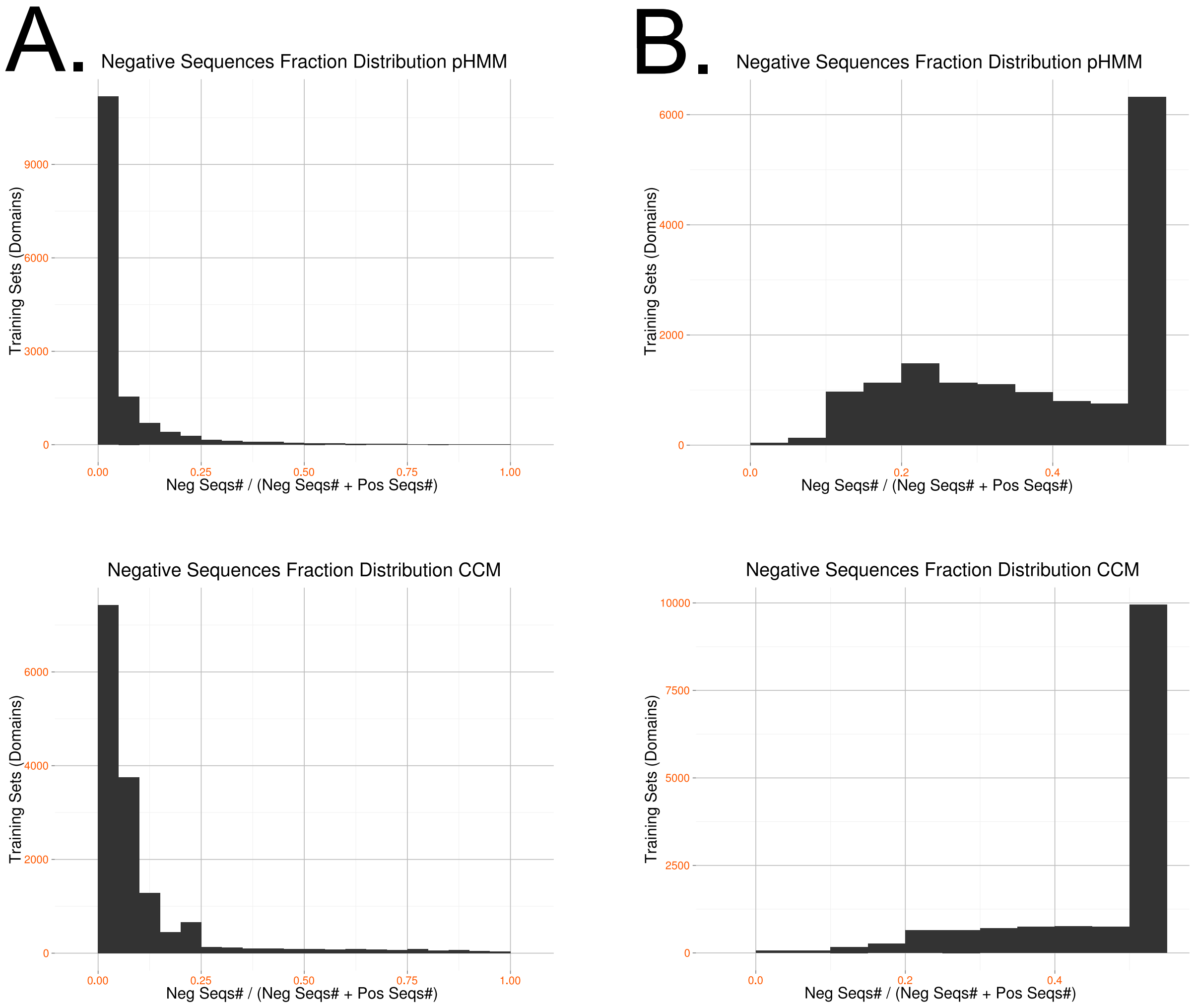

Figure S4: **Distribution of negative sequences generated or not by Markov models.** The impact of the construction of Markov models is illustrated by the distribution of negative sequences in the training sets of CCMs (bottom) and SCMs (pHMMs; top) constructed by either reshuffling of 2-mers or inversion only (**A**) compared to the generation of negative sequences constructed with the three methodological approaches, i.e. reshuffling, inversion and Markov models (**B**). Note that about 50% of the domains are characterised by training sets containing at least the 50% of negative sequences.

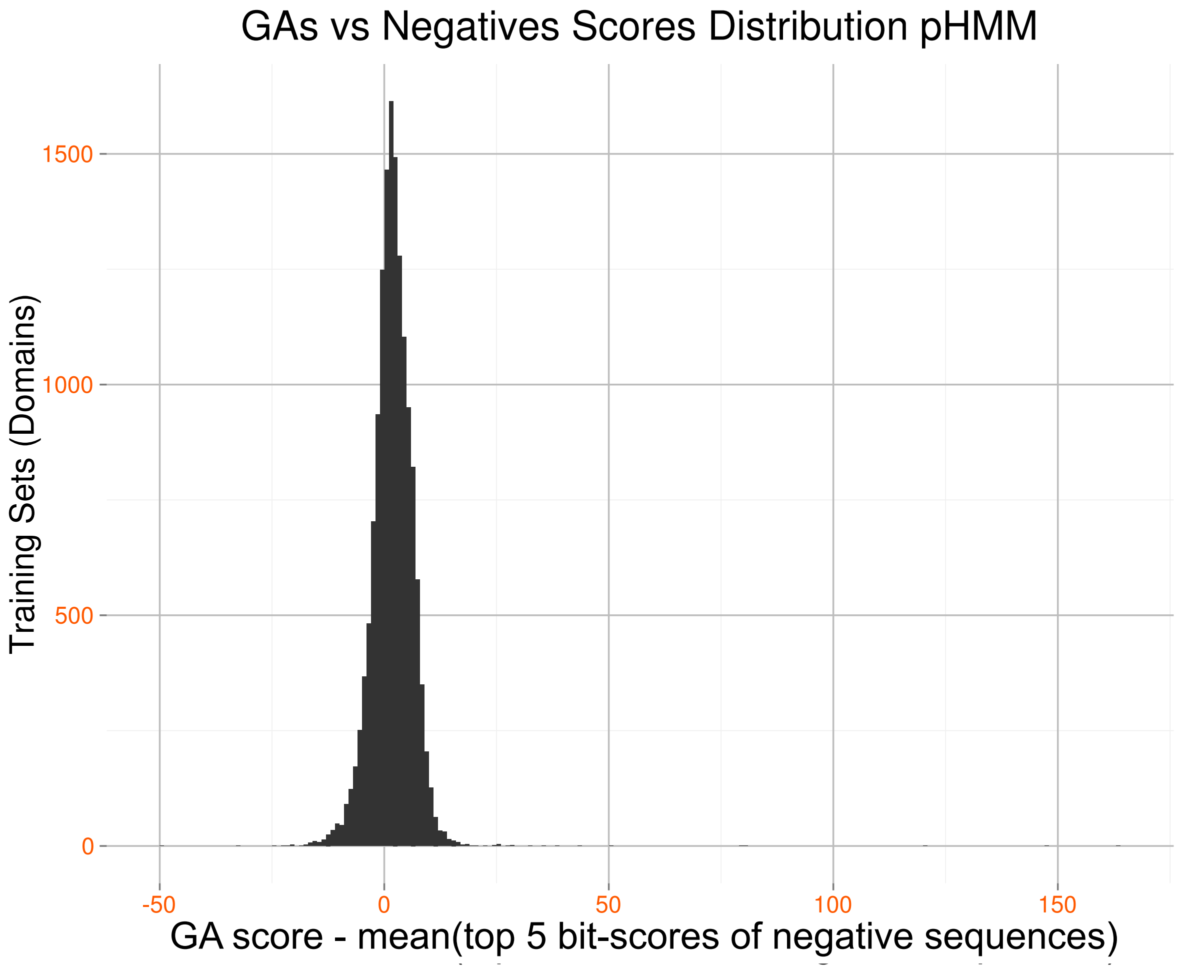

Figure S5: **GA vs negative bit-scores distribution.** For each Pfam domain and its associated SCM (pHMM), we computed the difference between the GA threshold associated to the SCM (pHMM) and the mean of the 5 best negative sequences bit-scores identified by the SCM (pHMM). The distribution of the differences shows a small standard deviation.

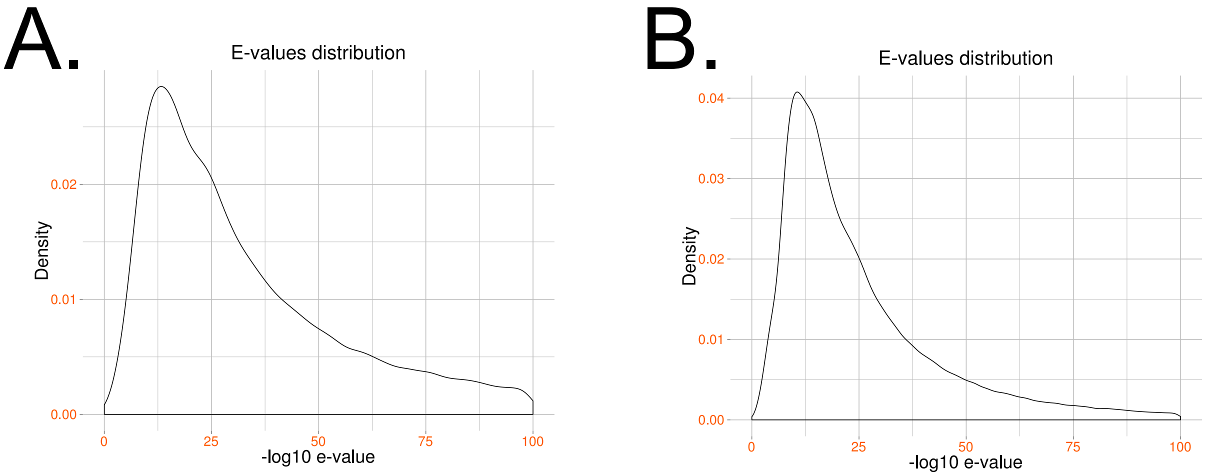

Figure S6: **Distribution of E-value for annotation based on Swiss-Prot and TrEMBL.** Distribution of E-values associated to the set of domains identified by MetaCLADE in the simulated metagenomic dataset. **A.** Annotation based on Swiss-Prot; **B.** annotation based on TrEMBL.

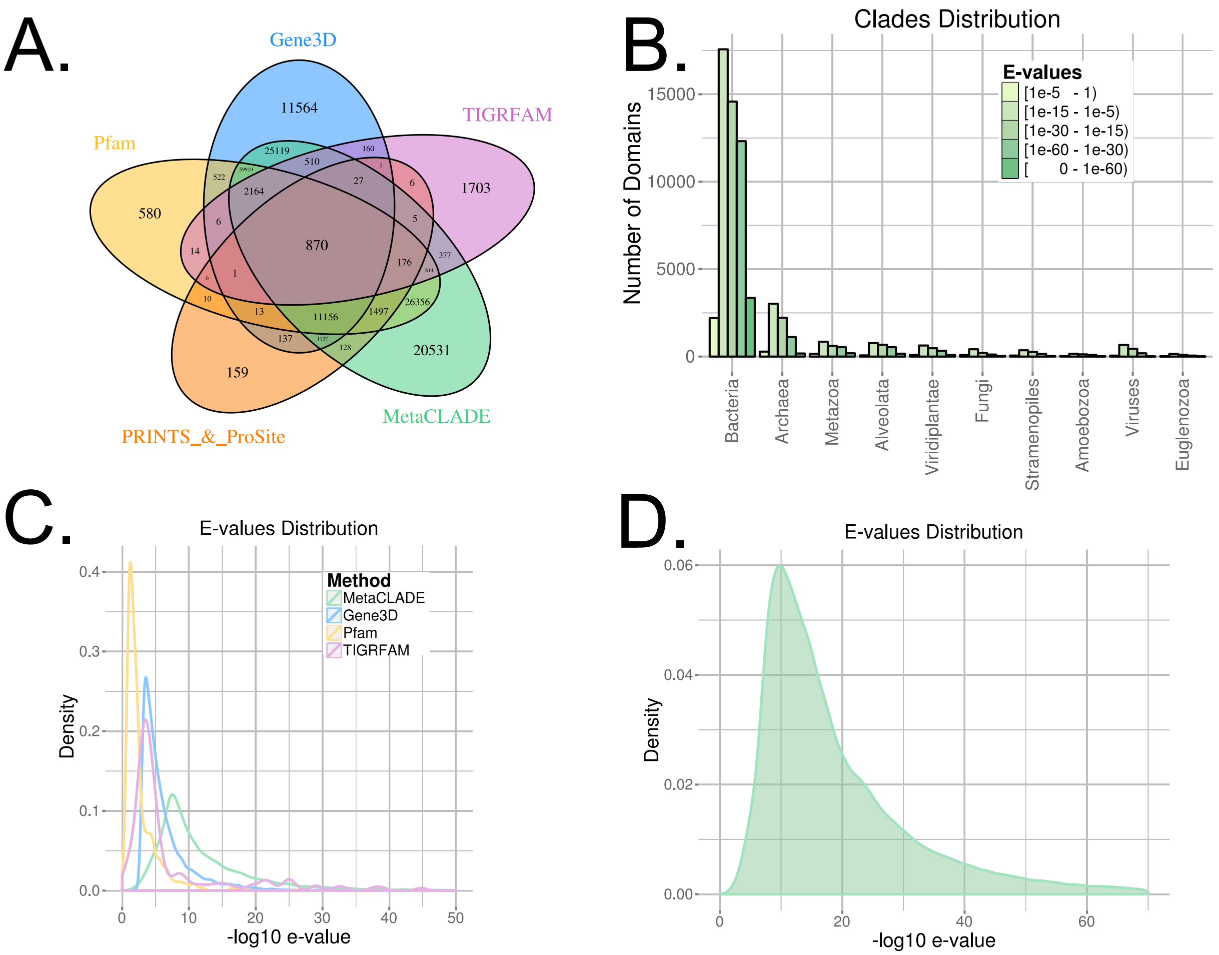

Figure S7: **Venn diagram of reads annotations for the metagenomic dataset of the Arctic Winter marine ecosystem.** **A.** Five domain annotation systems have been run on the Arctic Winter marine ecosystem metagenomic dataset: Pfam (yellow), Gene3D (blue), TIGRFAM (purple), PRINTS&ProSite (orange), and MetaCLADE (green). The number of reads annotated by one or several systems is reported. **B.** Distribution of species originating CCMs used to annotate the dataset. **C.** Distributions of E-values associated to the sets of domains identified in an exclusive manner by each tool. For instance, for MetaCLADE, we considered 20 531 domains. E-values are plot on the x-axis using a $-\log_{10}$ scale. **D.** Distributions of E-values associated to all domains identified by MetaCLADE. As in **C**, E-values are plot on the x-axis using a $-\log_{10}$ scale.

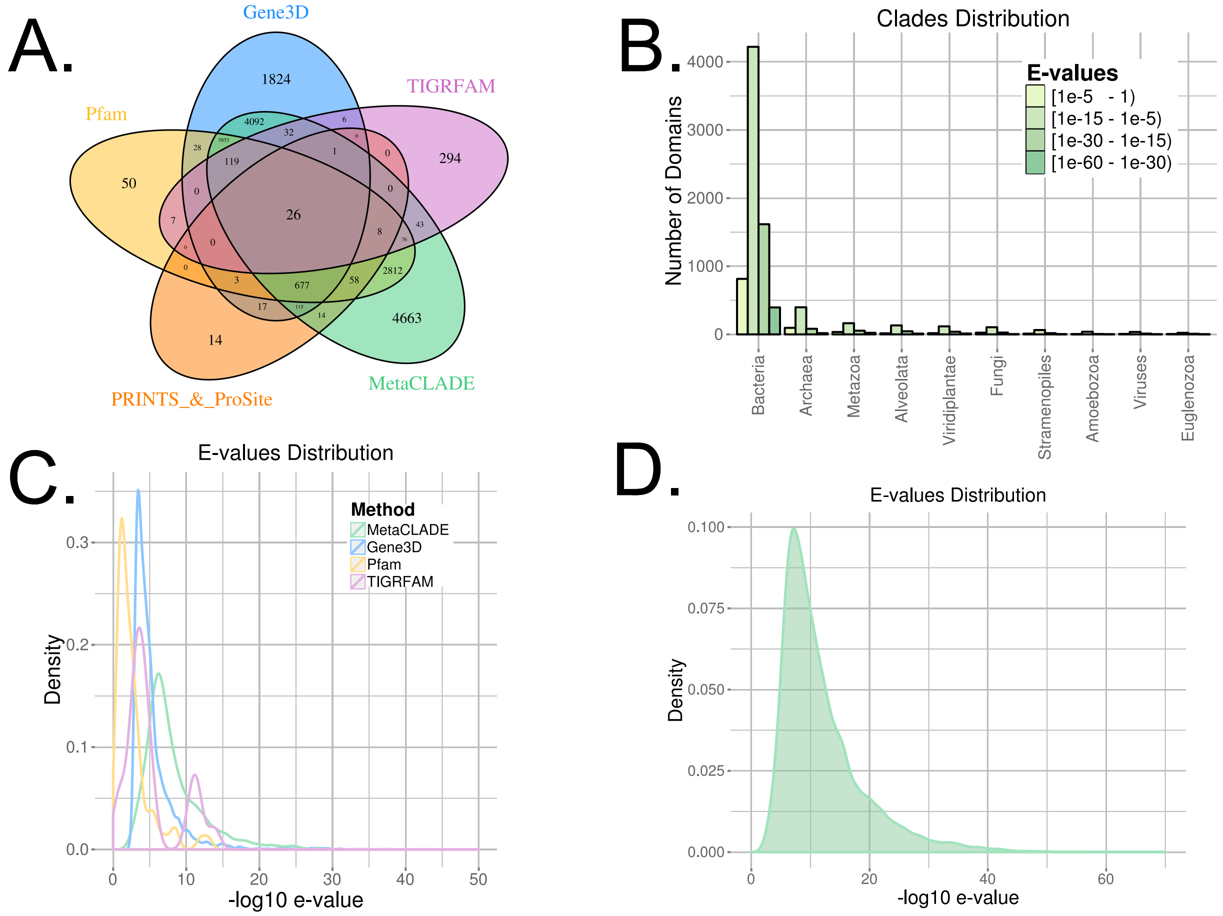

Figure S8: **Venn diagram of reads annotations for the Vindija Neanderthal metagenomic dataset.** **A.** Five domain annotation systems have been run on the Vindija Neanderthal metagenomic dataset: Pfam (yellow), Gene3D (blue), TIGRFAM (purple), PRINTS&ProSite (orange), and MetaCLADE (green). The number of reads annotated by one or several systems is reported. **B.** Distribution of species originating CCMs used to annotate the dataset. **C.** Distributions of E-values associated to the sets of domains identified in an exclusive manner by each tool. For instance, for MetaCLADE, we considered 4 663 domains. E-values are plot on the x-axis using a $-\log_{10}$ scale. **D.** Distributions of E-values associated to all domains identified by MetaCLADE. As in **C**, E-values are plot on the x-axis using a $-\log_{10}$ scale.

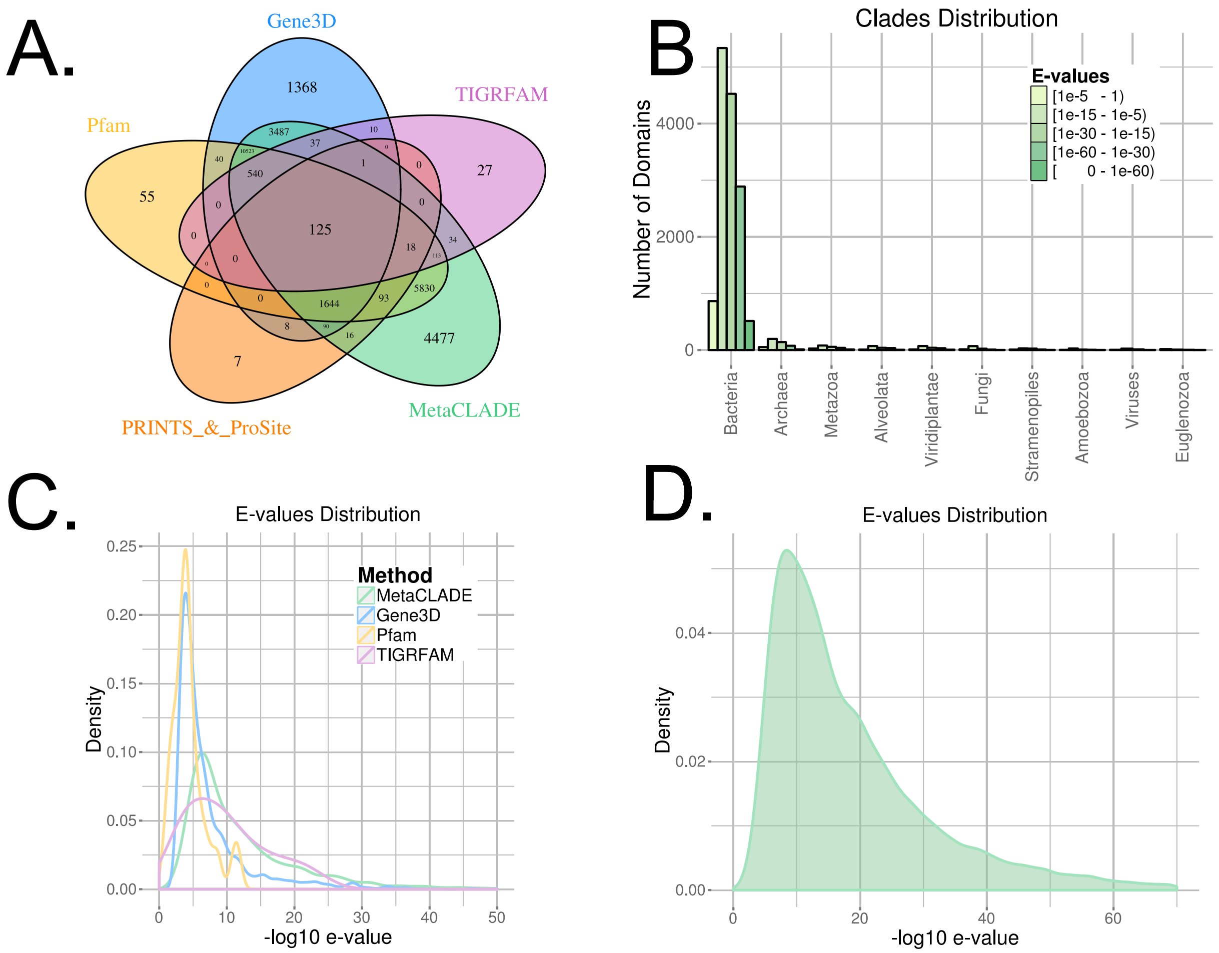

Figure S9: **Venn diagram of reads annotations for the human gut metagenome dataset. A.** Five domain annotation systems have been run on the human gut metagenome dataset: Pfam (yellow), Gene3D (blue), TIGRFAM (purple), PRINTS&ProSite (orange), and MetaCLADE (green). The number of reads annotated by one or several systems is reported. **B.** Distribution of species originating CCMs used to annotate the dataset. **C.** Distributions of E-values associated to the sets of domains identified in an exclusive manner by each tool. For instance, for MetaCLADE, we considered 4 477 domains. E-values are plot on the x-axis using a $-\log_{10}$ scale. **D.** Distributions of E-values associated to all domains identified by MetaCLADE. As in **C**, E-values are plot on the x-axis using a $-\log_{10}$ scale.

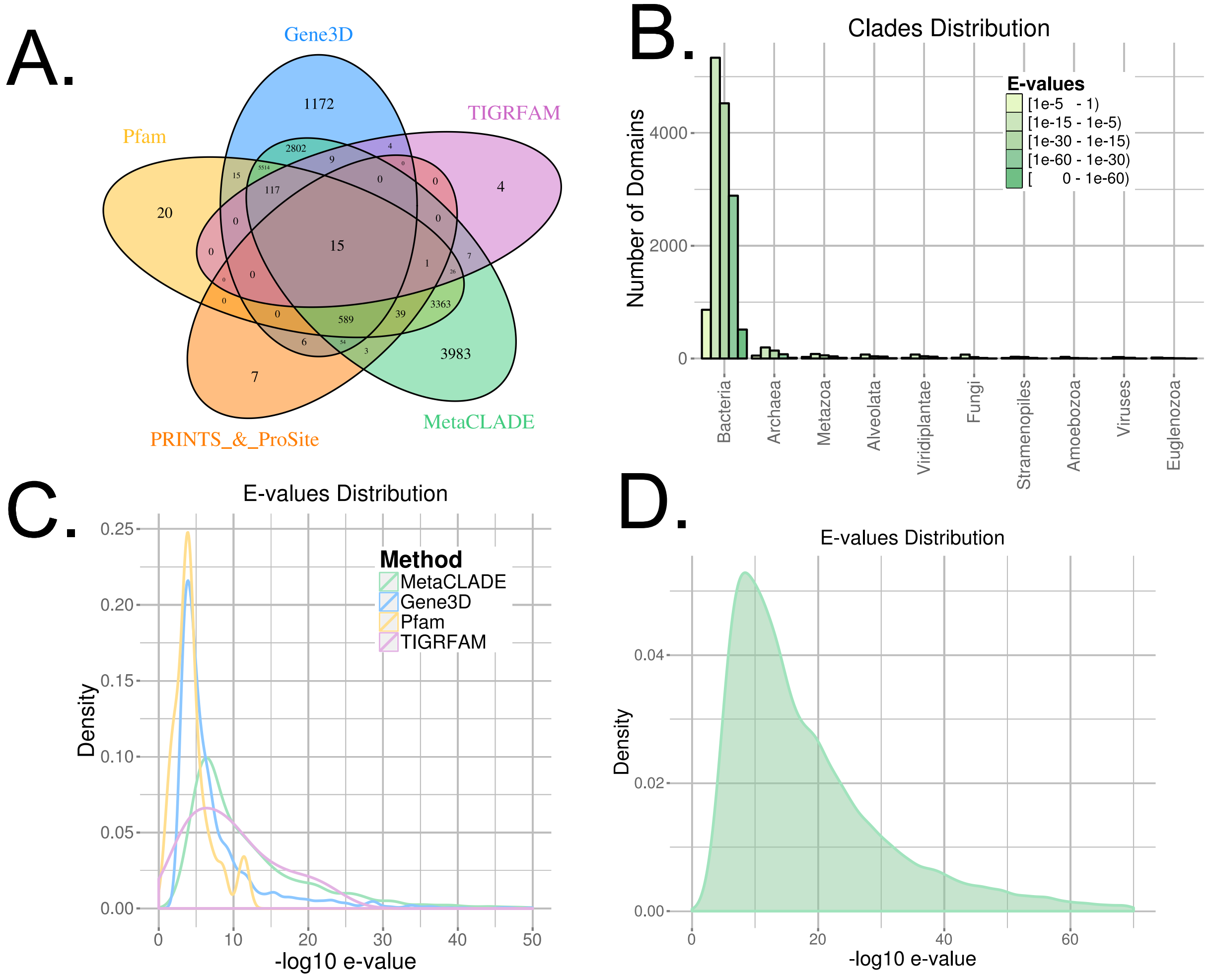

Figure S10: **Venn diagram of reads annotations for the human gut metatranscriptomic dataset. A.** Five domain annotation systems have been run on the human gut metatranscriptomic dataset: Pfam (yellow), Gene3D (blue), TIGRFAM (purple), PRINTS&ProSite (orange), and MetaCLADE (green). The number of reads annotated by one or several systems is reported. **B.** Distribution of species originating CCMs used to annotate the dataset. **C.** Distributions of E-values associated to the sets of domains identified in an exclusive manner by each tool. For instance, for MetaCLADE, we considered 3 983 domains. E-values are plot on the x-axis using a $-\log_{10}$ scale. **D.** Distributions of E-values associated to all domains identified by MetaCLADE. As in **C**, E-values are plot on the x-axis using a $-\log_{10}$ scale.

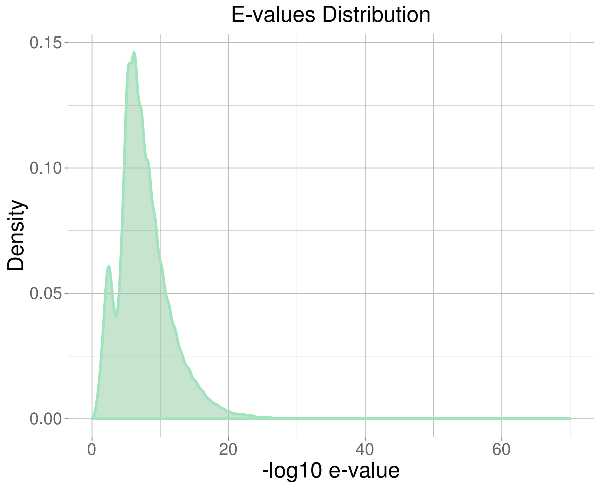

Figure S11: **Distribution of E-values for the O'Connor lake metagenomic dataset.** Distribution of E-values associated to all domains identified by MetaCLADE. E-values are plot on the x-axis using a $-\log_{10}$ scale. Note that this distribution cannot be immediately compared to **Figures S7D-S10D** and **Figure 5D** because it corresponds only to domain identifications that were not identified before. The detection of these hits constitutes a challenge.

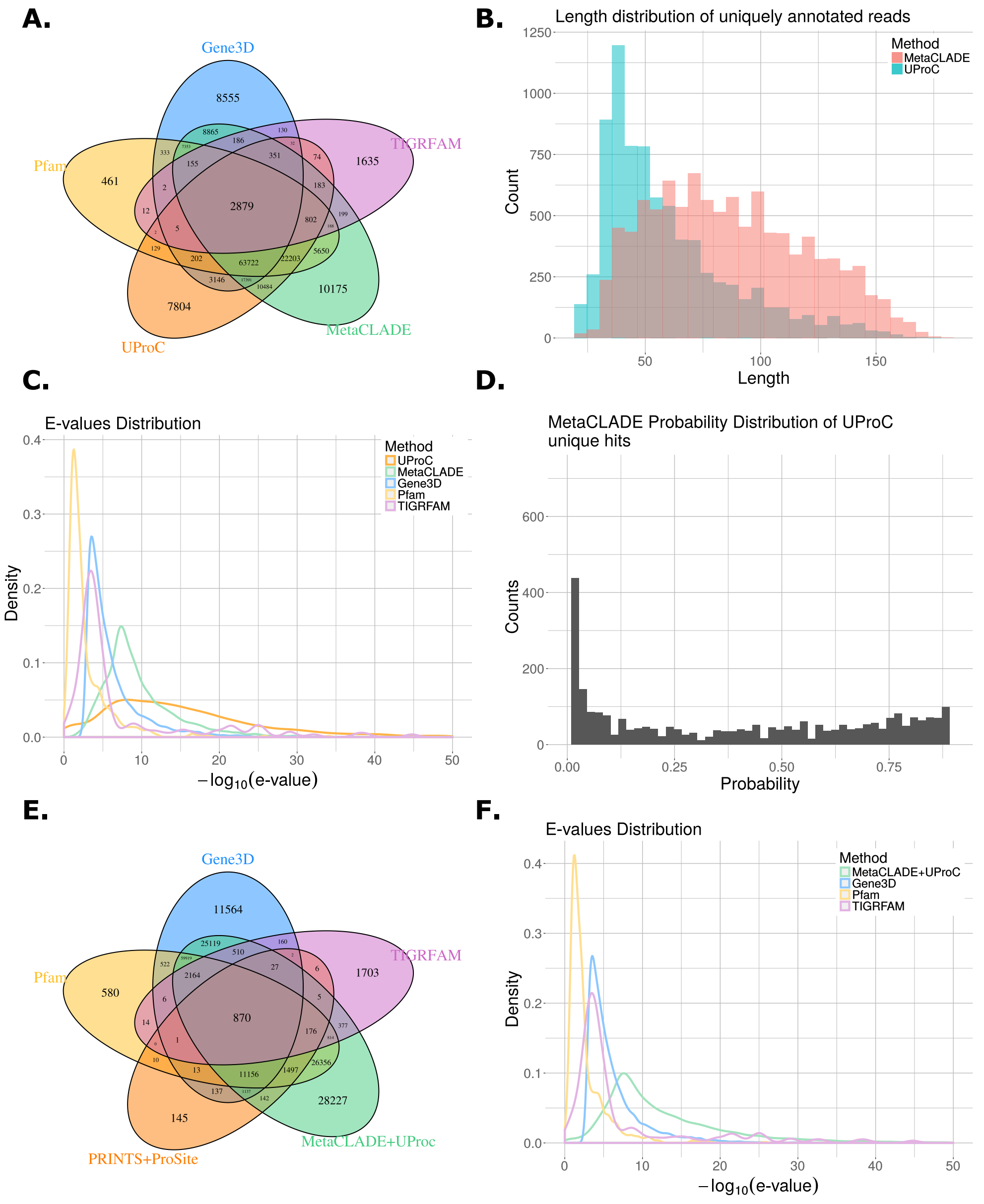

Figure S12: **Venn diagram of reads annotations obtained with UProC, InterProScan and MetaCLADE. A.** Five domain annotation systems were run on the **Arctic Winter marine ecosystem metagenomic** dataset: Pfam (yellow), Gene3D (blue), TIGRFAM (purple), UProC (orange), and MetaCLADE (green). The number of reads annotated by one or several systems is reported. **B.** Length distribution of reads annotated exclusively by either UProC or MetaCLADE. **C.** Distributions of E-values associated to the sets of domains identified in an exclusive manner by each tool. For instance, for MetaCLADE, we considered 22 059 domains (see **A**). E-values are plotted on the x-axis using a $-\log_{10}$ scale. **D.** Distribution of probabilities associated to those exclusive UProC domain annotations that have been detected by MetaCLADE but discarded because of the probability threshold 0.9. **E** Venn diagram as in **A** of **Figure S7**, but where MetaClade is replaced by MetaClade+UProC. **F** As in **C**, but where MetaClade is replaced by MetaClade+UProC.

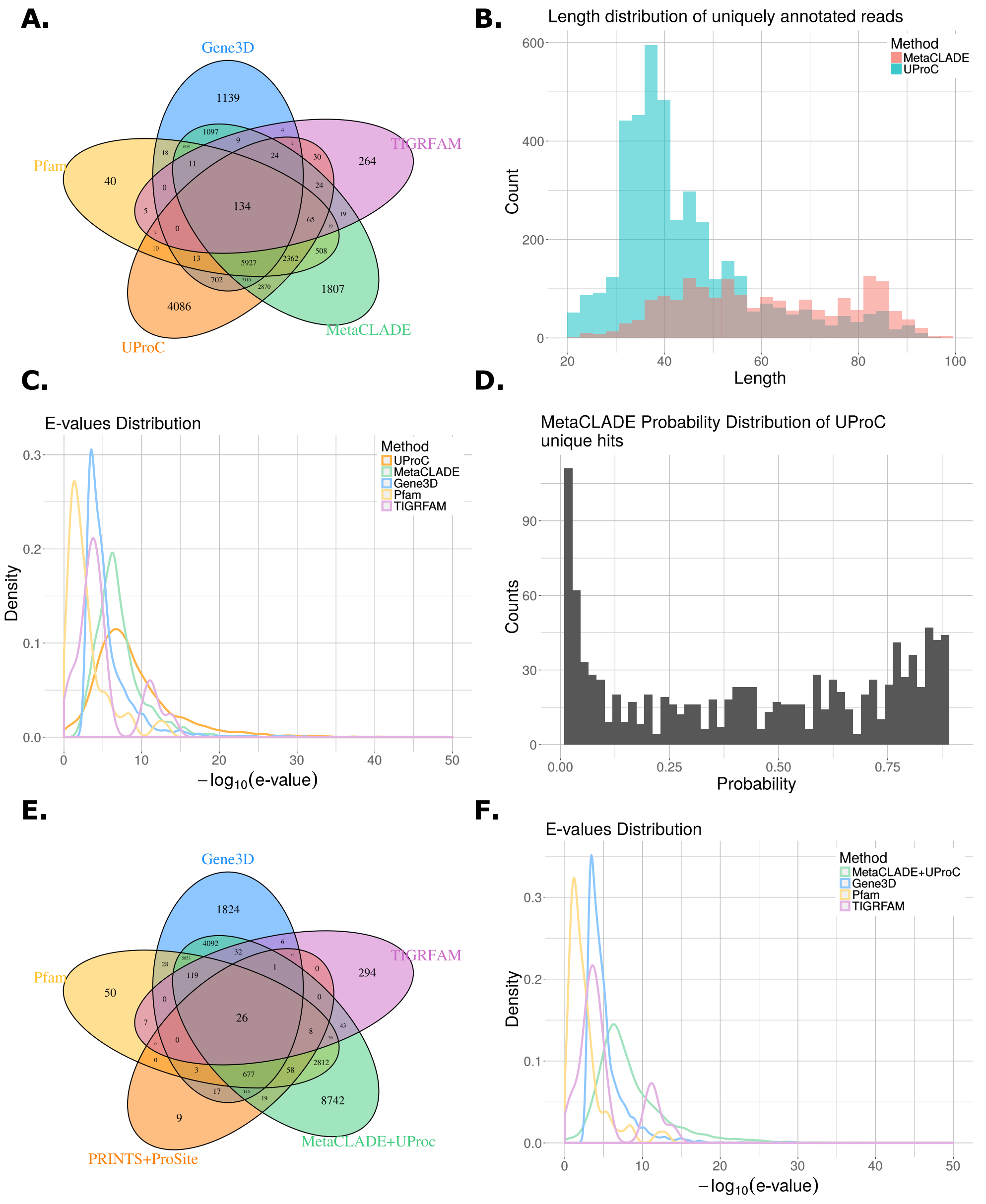

Figure S13: **Venn diagram of reads annotations obtained with UProC, InterProScan and MetaCLADE. A.** Five domain annotation systems were run on the **Bone sample from Vindjia Neaderthal metagenomic** dataset: Pfam (yellow), Gene3D (blue), TIGRFAM (purple), UProC (orange), and MetaCLADE (green). The number of reads annotated by one or several systems is reported. **B.** Length distribution of reads annotated exclusively by either UProC or MetaCLADE. **C.** Distributions of E-values associated to the sets of domains identified in an exclusive manner by each tool. For instance, for MetaCLADE, we considered 22 059 domains (see **A**). E-values are plotted on the x-axis using a $-\log_{10}$ scale. **D.** Distribution of probabilities associated to those exclusive UProC domain annotations that have been detected by MetaCLADE but discarded because of the probability threshold 0.9. **E** Venn diagram as in **A** of **Figure S8**, but where MetaClade is replaced by MetaClade+UProC. **F** As in **C**, but where MetaClade is replaced by MetaClade+UProC.

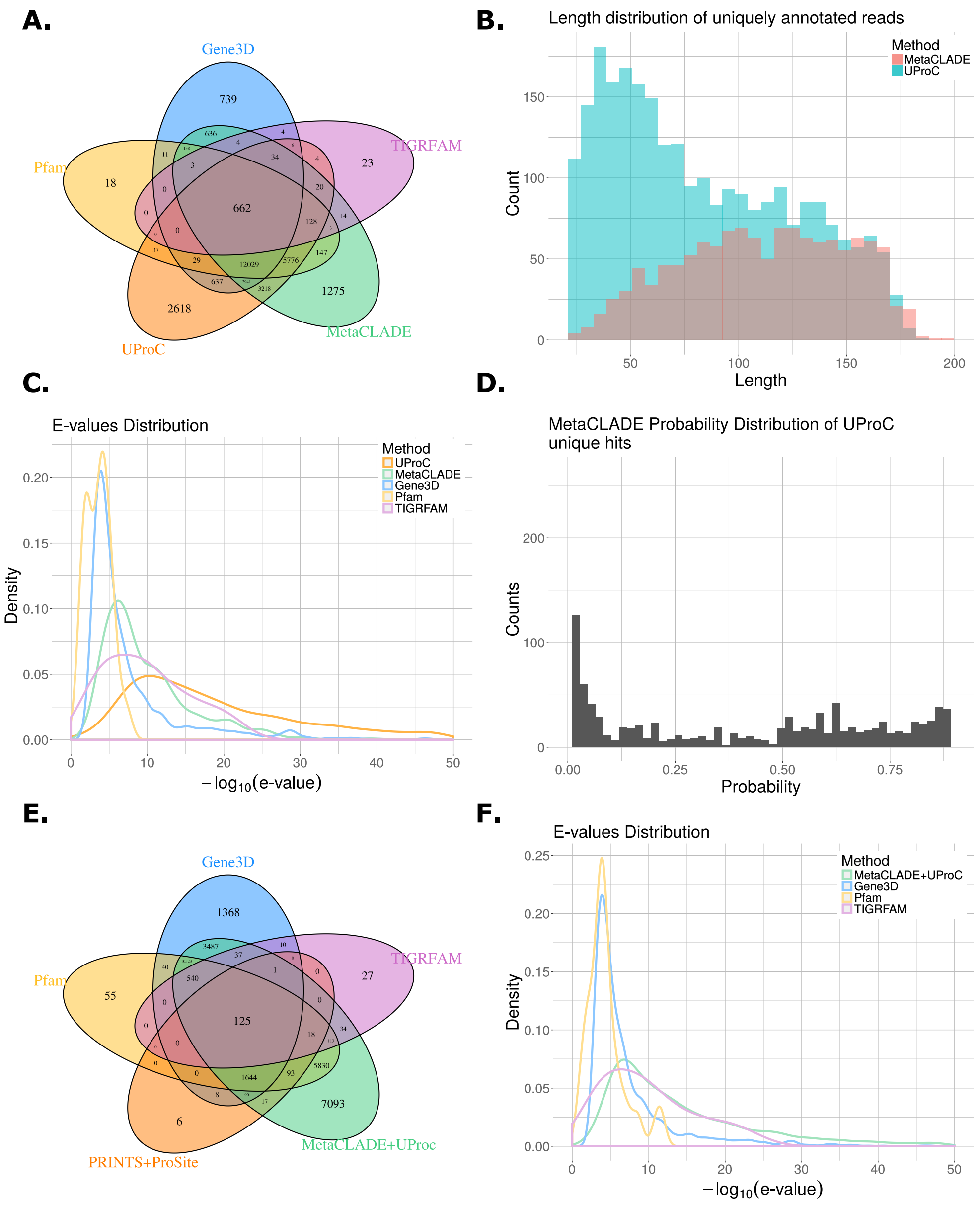

Figure S14: **Venn diagram of reads annotations obtained with UProC, InterProScan and MetaCLADE. A.** Five domain annotation systems were run on the **Human gut metagenomic** dataset: Pfam (yellow), Gene3D (blue), TIGRFAM (purple), UProC (orange), and MetaCLADE (green). The number of reads annotated by one or several systems is reported. **B.** Length distribution of reads annotated exclusively by either UProC or MetaCLADE. **C.** Distributions of E-values associated to the sets of domains identified in an exclusive manner by each tool. For instance, for MetaCLADE, we considered 22 059 domains (see **A**). E-values are plotted on the x-axis using a $-\log_{10}$ scale. **D.** Distribution of probabilities associated to those exclusive UProC domain annotations that have been detected by MetaCLADE but discarded because of the probability threshold 0.9. **E** Venn diagram as in **A** of **Figure S9**, but where MetaClade is replaced by MetaClade+UProC. **F** As in **C**, but where MetaClade is replaced by MetaClade+UProC.

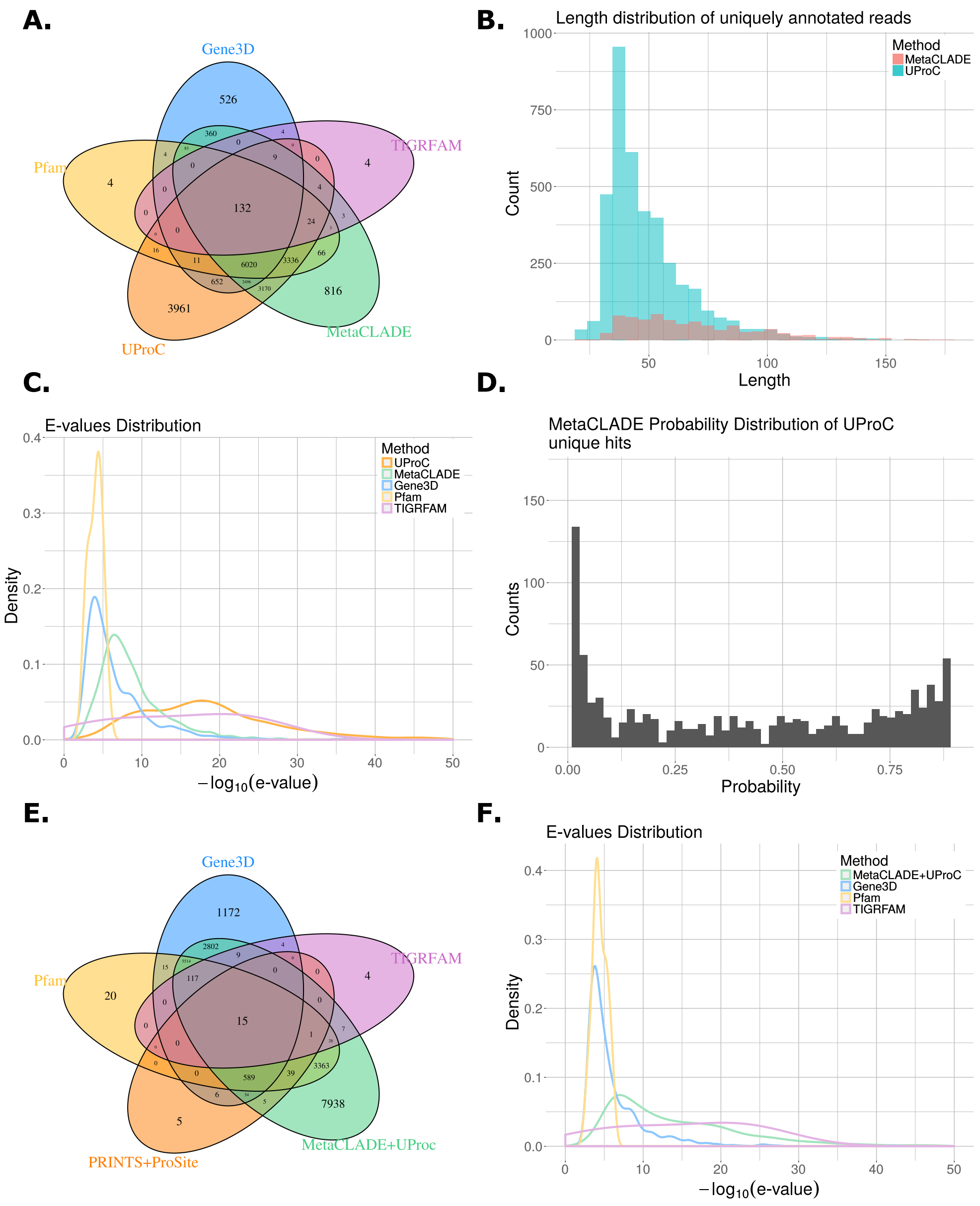

Figure S15: **Venn diagram of reads annotations obtained with UProC, InterProScan and MetaCLADE. A.** Five domain annotation systems were run on the **Human gut metatranscriptomic** dataset: Pfam (yellow), Gene3D (blue), TIGRFAM (purple), UProC (orange), and MetaCLADE (green). The number of reads annotated by one or several systems is reported. **B.** Length distribution of reads annotated exclusively by either UProC or MetaCLADE. **C.** Distributions of E-values associated to the sets of domains identified in an exclusive manner by each tool. For instance, for MetaCLADE, we considered 22 059 domains (see **A**). E-values are plotted on the x-axis using a $-\log_{10}$ scale. **D.** Distribution of probabilities associated to those exclusive UProC domain annotations that have been detected by MetaCLADE but discarded because of the probability threshold 0.9. **E** Venn diagram as in **A** of **Figure S10**, but where MetaClade is replaced by MetaClade+UProC. **F** As in **C**, but where MetaClade is replaced by MetaClade+UProC.

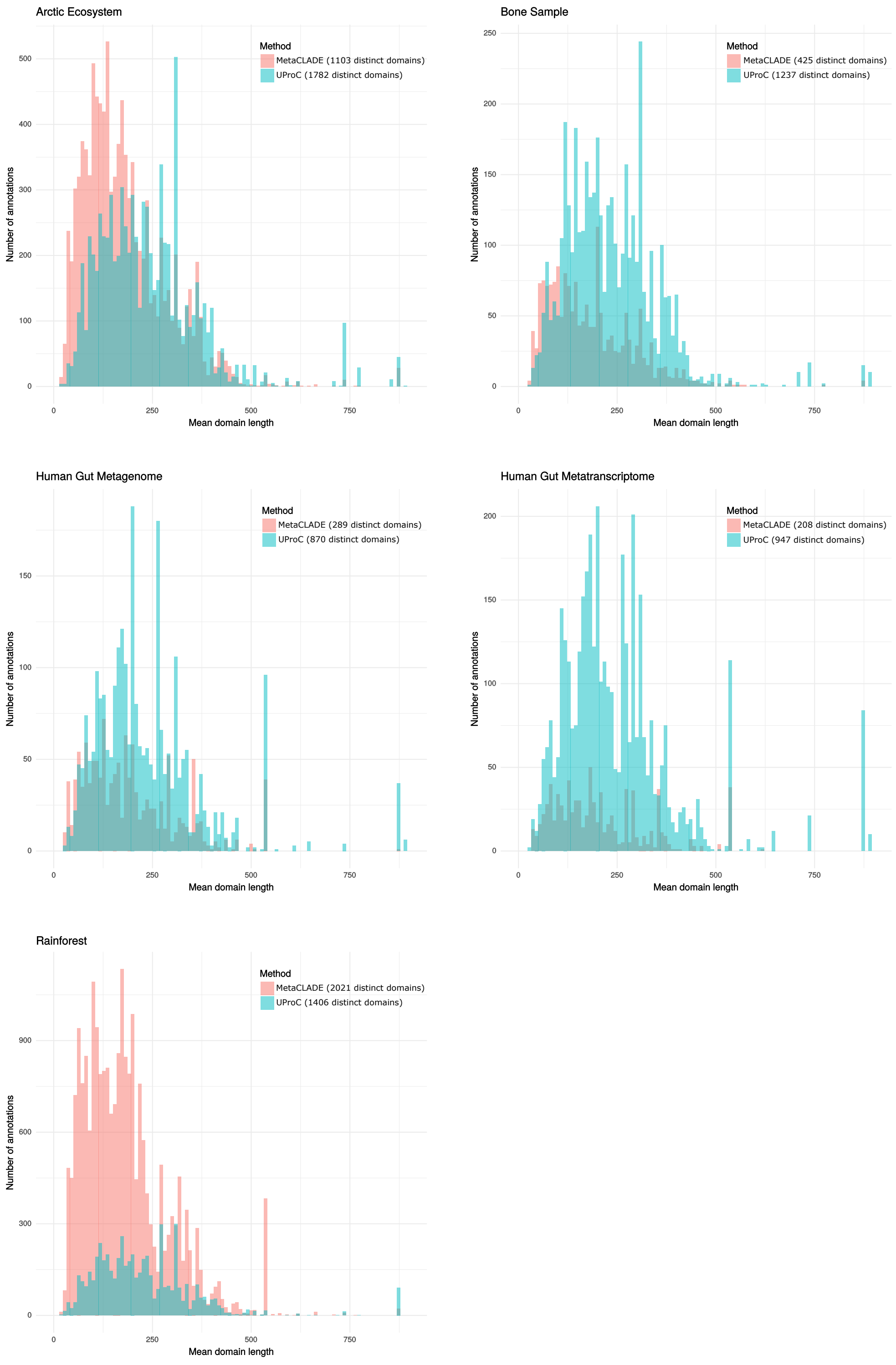

Figure S16: **Length distribution of domains identified in an exclusive manner by UProC and MetaCLADE.** The five MG/MT datasets in **Table 1** are analysed with UProC and MetaCLADE. In each plot, the number of annotations provided by MetaCLADE/UProC in an exclusive manner is reported with respect to domain length. The number of distinct domains identified by the annotations of a method on a specific dataset is reported in the legend of the plot. From the distributions, one can observe that MetaCLADE seems to prefer shorter domains compared to UProC.

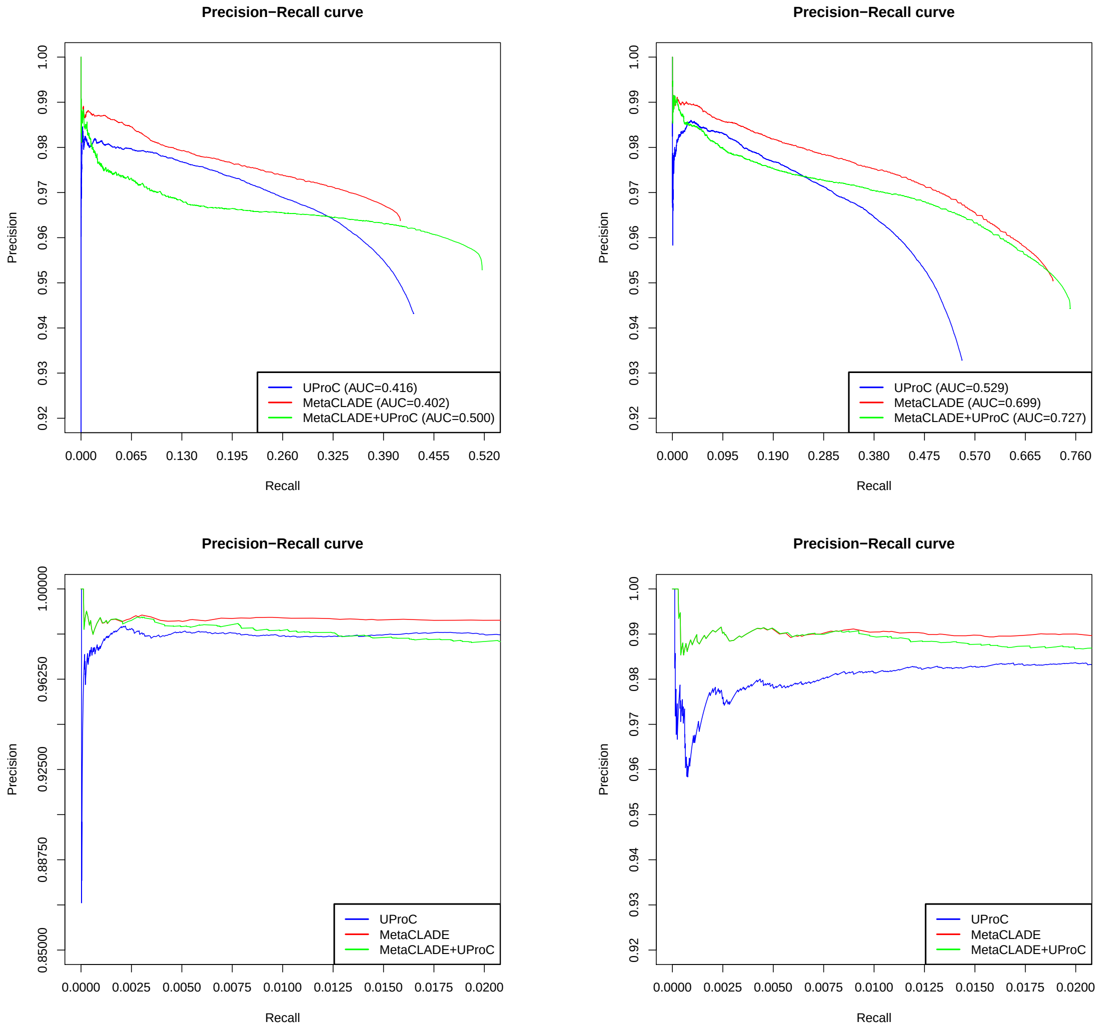

Figure S17: **Precision-recall plots of UProC, MetaCLADE and MetaCLADE+UProC on the datasets generated from Guerrero Negro Hypersaline microbial Mat project - 100bp and 200bp reads, with CLADE as ground truth**. **Top:** analyses of the datasets with 100bp (**left**) and 200bp (**right**) long reads. The curves are computed by considering CLADE (version 27) annotation as ground truth and an equivalence of domain annotation for domain pairs belonging to the same clan. For each curve, the last point on the right corresponds to the value indicated in Table 3 of the article. Also, for each curve, the associated AUC is reported in parenthesis on the inset legend. **Bottom:** zooms of the curves displayed on the top on low Recall values.

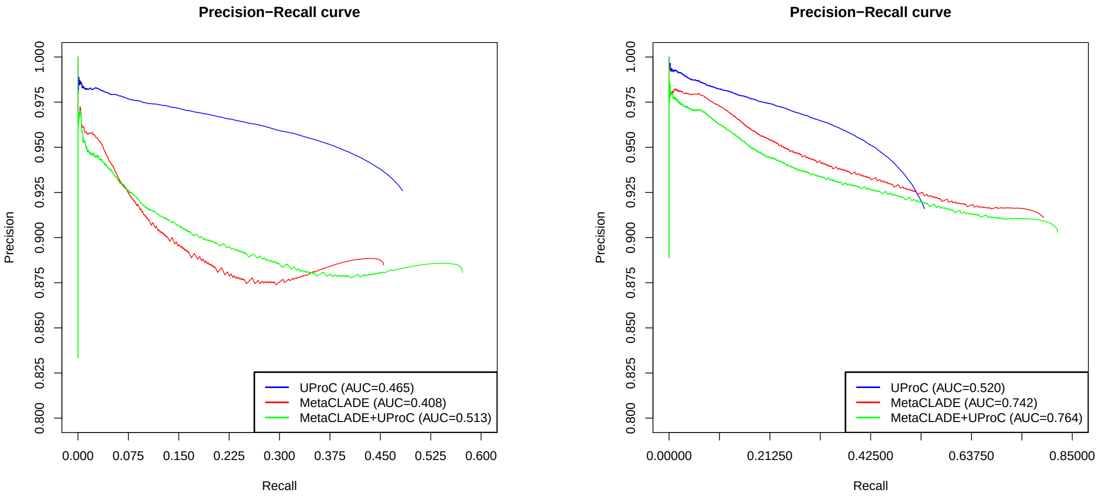

Figure S18: **Precision-recall plots of UProC, MetaCLADE and MetaCLADE+UProC on the datasets generated from Guerrero Negro Hypersaline microbial Mat project - 100bp and 200bp reads, with Pfam27 as ground truth**. Analyses of the datasets with 100bp (**left**) and 200bp (**right**) long reads. The curves are computed by considering Pfam (version 27) annotation as ground truth and an equivalence of domain annotation for domain pairs belonging to the same clan. For each curve, the last point on the right corresponds to the value indicated in Table 3 of the article. Also, for each curve, the associated AUC is reported in parenthesis on the inset legend.

Table S1: **Ranking scores for selecting hits on the third step of domain selection**

CCM		SCM	
**bit-score (≥)**	**significance**	**bit-score (≥)**	**significance**
90	1	40	1
80	.9	35	.9
70	.8	30	.8
60	.7	25	.7
50	.6	23	.6
40	.5	20	.5
30	.4	18	.4
25	.3	15	.3
20	.2	12	.2
0	.1	0	.1

The ranking score is computed based on the significance of the bit-score (value between 0 and 1), for CCMs (left) and SCMs (right). Values on the table are the ones giving best results on the dataset simulated from 56 fully sequences genomes.

Table S2: **Archaeal and bacterial genomes used for generating simulated metagenomic data**

Organism	NCBI Accession Number	Size (Mbp)
Aeropyrum pernix K1	BA000002	1,66
Archaeoglobus fulgidus DSM 4304	AE000782	2,17
Methanocaldococcus jannaschii DSM 2661	L77117	1,66
Methanococcus maripaludis	BX950229	1,66
Methanopyrus kandleri AV19	AE009439	1,69
Methanosarcina acetivorans C2A	AE010299	5,75
Nanoarchaeum equitans Kin4-M	AE017199	0,49
Pyrobaculum aerophilum str. IM2	AE009441	2,22
Pyrococcus abyssi GE5	AL096836	1,76
Pyrococcus furiosus DSM 3638	AE009950	1,90
Pyrococcus horikoshii OT3	BA000001	1,73
Candidatus Pelagibacter ubique HTCC1062	CP000084	1,30
Colwellia psychrerythraea 34H	CP000083	5,37
Desulfotalea psychrophila LSv54	CR522870	3,52
Erythrobacter litoralis HTCC2594	CP000157	3,05
Geobacillus kaustophilus HTA426	BA000043	3,54
Hahella chejuensis KCTC 2396	CP000155	7,21
Hyphomonas neptunium ATCC 15444	CP000158	3,70
Idiomarina loihiensis L2TR	AE017340	2,83
Jannaschia sp. CCS1	CP000264	4,31
Magnetococcus marinus MC-1	CP000471	4,71
Maricaulis maris MCS10	CP000449	3,36
Oceanobacillus iheyensis HTE831	BA000028	3,63
Photobacterium profundum	CR354531	4,08
Polaromonas sp. JS666	CP000316	5,20
Prochlorococcus marinus str. AS9601	CP000551	1,66
Prochlorococcus marinus str. MIT 9301	CP000576	1,64
Prochlorococcus marinus str. MIT 9303	CP000554	2,68
Prochlorococcus marinus str. MIT 9515	CP000552	1,70
Prochlorococcus marinus MIT9313 complete genome	BX548175	2,41
Prochlorococcus marinus str. NATL1A	CP000553	1,86
Prochlorococcus marinus str. NATL2A	CP000095	1,84
Pseudoalteromonas atlantica T6c	CP000388	5,18
Pseudoalteromonas haloplanktis TAC125	CR954246	3,21
Pseudoalteromonas haloplanktis TAC125	CR954247	0,63
Psychromonas ingrahamii 37	CP000510	4,55
Rhodopirellula baltica SH 1	BX119912	7,14
Roseobacter denitrificans OCh 114	CP000362	4,13
Saccharophagus degradans 2-40	CP000282	5,05
Shewanella denitrificans OS217	CP000302	4,54
Shewanella frigidimarina NCIMB 400	CP000447	4,84
Shewanella sp. MR-4	CP000446	4,70
Shewanella sp. MR-7	CP000444	4,79
Shewanella loihica PV-4	CP000606	4,60
Shewanella sp. W3-18-1	CP000503	4,70
Ruegeria pomeroyi DSS-3	CP000031	4,10
Ruegeria sp. TM1040	CP000377	3,20
Sphingopyxis alaskensis RB2256	CP000356	3,34
Synechococcus sp. WH 8102	BX548020	2,43
Synechococcus sp. CC9311	CP000435	2,60
Synechococcus sp. CC9605	CP000110	2,51
Synechococcus sp. CC9902	CP000097	2,23
Thermotoga maritima MSB8	CP004077	1,86
Trichodesmium erythraeum IMS101	CP000393	7,75
Vibrio fischeri ES114 chromosome I	CP000020	2,89
Vibrio fischeri ES114 chromosome II	CP000021	1,33

11 archaeal (top) and 44 bacterial (bottom) genomes, NCBI accession numbers and sizes (in Mbp)

Table S3: **Comparison of MetaCLADE and HMMer on the simulated 454 dataset (ground truth: Swiss-Prot annotation of 65 816 domains).**

Tools	Ground truth	Clan-oriented						No Clan					
	annotations	TP	FP	FN	Recall	Precision	F-score	TP	FP	FN	Recall	Precision	F-score
MetaCLADE	65 816	56 763	1 594	52	99.91	97.27	98.57	54 833	3 524	1 982	96.51	93.96	95.22
HMMer	65 816	49 243	430	7	99.99	99.13	99.56	48 552	1 123	698	98.58	97.74	98.16

Table S4: **Characteristics of the metagenomic/metatranscriptomic datasets**

Datasets	Data type	Number of reads	Reads mean size (nt)	Sequencing technique
Puerto Rico Rainforest soil	metaG	790 804	530	454 GS FLX Titanium
Arctic Winter marine ecosystem	metaG	360 911	261	454 GS FLX Titanium
Bone sample from Vindija Neanderthal	metaG	83 699	160	454 GS FLX Titanium
Human gut metagenomics	metaG	40 722	381	454 GS FLX Titanium
Human gut metatranscriptomics	metaT	84 215	171	454 GS FLX Titanium
Antarctic (ANT)	metaT	268 208	196	454 GS FLX Titanium
Arctic (ARC)	metaT	427 315	259	454 GS FLX Titanium
Equatorial Pacific (EPAC)	metaT	258 976	166	454 GS FLX Titanium
North Pacific (NPAC)	metaT	271 357	269	454 GS FLX Titanium
North Atlantic (NATL)	metaT	401 888	294	454 GS FLX Titanium
O'Connor lake sample	metaG	1 315 435	123	Illumina HiSeq 2000
MG from 56 genomes (simulated)	metaG	500 000	523	454 GS FLX Titanium simulated with FlowSim**
MG from 23 marine genomes (simulated)	metaG	1 955 009*	100	Illumina-like simulated with WGSIM**
GNHM - 100bp (simulated)	metaG	1 243 889	100	exact fragments from Sanger sequencing
GNHM - 200bp (simulated)	metaG	683 669	200	exact fragments from Sanger sequencing

* Number of predicted CDS.

** 1% error rate.

Table S5: **Download sites for the metagenomic/metatranscriptomic datasets**

Datasets	Download site
Puerto Rico Rainforest soil	www.ebi.ac.uk/metagenomics/projects/SRP001743/samples/ SRS009922/runs/SRR034257,SRR034258/results/versions/1.0#ui-id-8
Arctic Winter marine ecosystem	https://www.ebi.ac.uk/metagenomics/projects/ERP001178/samples/ ERS089005/runs/ERR071289/results/versions/1.0
Bone sample from Vindija Neanderthal	www.ebi.ac.uk/metagenomics/projects/ERP002047/samples/ ERS198355/runs/ERR208325/results/versions/1.0#ui-id-8
Human gut metagenomics	www.ebi.ac.uk/metagenomics/projects/ERP001506/samples/ ERS149129/runs/ERR139689/results/versions/2.0#ui-id-8
Human gut metatranscriptomics	www.ebi.ac.uk/metagenomics/projects/ERP001506/samples/ ERS149135/runs/ERR139701/results/versions/2.0#ui-id-8
O'Connor lake sample	https://www.ebi.ac.uk/metagenomics/projects/ERP009498/samples/ ERS656878/runs/ERR753910/results/versions/2.0#ui-id-10
MG from 56 marine genomes (simulated)	http://www.lcqb.upmc.fr/metaclade/databases/Simulated.tar.gz
MG from 23 marine genomes (simulated)	http://www.lcqb.upmc.fr/metaclade/databases/SimMarine.tar.gz https://sourceforge.net/projects/hmm-graspx/files/SupplementaryData/SimMarine_23G.fa.tgz
GNHM - 100bp/200bp (simulated)	http://www.lcqb.upmc.fr/metaclade/databases/GNHM.tar.gz

Table S6: **Most abundant new domains identified in the O'Connor lake metagenomic dataset**

Pfam Acc	IPR	Matches from InterProScan	Additional matches by MetaCLADE	Description
PF00528	IPR000515	1 578	1109	ABC transporter type 1
PF00873	IPR001036	961	1093	Acriflavin resistance protein
PF00005	IPR003439	2087	1081	ABC transporter-like
PF12704	IPR025857	121	878	MacB-like periplasmic core domain (found in a variety of ABC transporters)
PF07690	IPR011701	64	734	Major facilitator superfamily (transporter family)
PF02653	IPR001851	374	617	ABC transporter, permease
PF00593	IPR000531	633	464	TonB-dependent receptor, beta-barrel
PF00501	IPR000873	1156	458	AMP-dependent synthetase/ligase
PF13520	IPR002293	29	437	Amino acid/polyamine transporter
PF01609	IPR002559	40	396	Transposase, IS4-like

Table S7: **Conserved motifs used to identify new domains in the O'Connor lake metagenomic dataset**

Motif number	Conserved motif logo	E-value	Sites	Width
motif 1	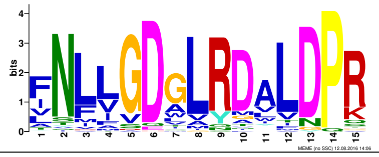	3.8e-367	59	15
motif 2	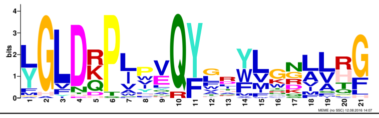	3.1e-236	37	21
motif 3	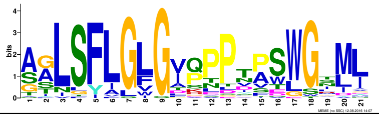	1.0e-233	39	21
motif 4	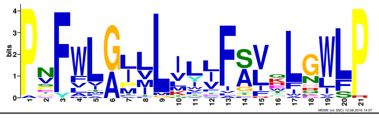	1.4e-147	30	21
motif 5	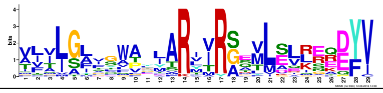	1.4e-107	23	29
motif 6	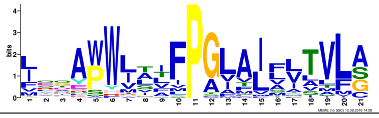	7.1e-108	34	21
motif 7	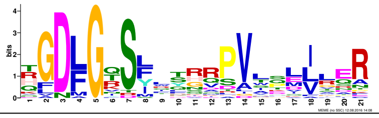	1.1e-104	29	21
motif 8	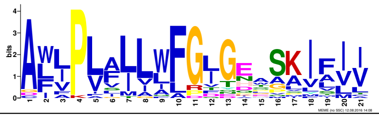	1.2e-91	27	21
motif 9	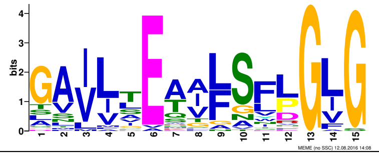	1.3e-78	36	15
motif 10	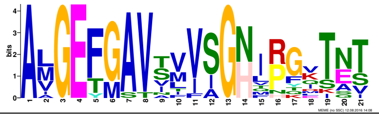	1.7e-76	14	21

Table S8: **Computational time for the analysis of the datasets (using one core)**

Sample	Domain hit identification	First filter	Second filter	Third filter	Total time for hit selection	Total number of amino acids in predicted CDS
Puerto Rico Rain Forest soil metagenome	3 354.35 h	183.24 h	48.76 h	19.66 m	3 586.67 h	69 362 268
Arctic Winter marine ecosystem	851.11 h	112.61 h	26.71 h	10 m	990.59 h	28 532 446
Bone sample from Vindija Neanderthal	146.72 h	8.52 h	5.81 h	5.98 m	161.14 h	3 745 301
Human gut metagenome	113.2 h	6.49 h	4.46 h	5.89 m	124.24 h	1 878 455
Human gut metatranscriptome	98.4 h	7.37 h	5.07 h	5.85 m	110. 93 h	1 230 998
O'Connor lake metagenome	1 089.40 h	94.3 h	5.23 h	8.91 m	1 189.07 h	36 505 990
MG from 56 genomes (simulated)	3 758.21 h	137.52 h	48.87 h	12.43 m	3 944.80 h	77 903 127
MG from 23 marine genomes (simulated)	732.42 h	41.73 h	1.55 h	4.09 m	775.76 h	6 270 236
GNHM - 100bp (simulated)						
Pfam ground truth	1 573.91 h	54.21 h	1.45 h	29.84 s	1 629.58 h	40 011 461
CLADE ground truth	1 023.3 h	38.39 h	1.11 h	6.68 m	1 062.91 h	39 633 479
GNHM - 200bp (simulated)						
Pfam ground truth	616.40 h	87.13 h	1.56 h	54.10 s	705.12 h	44 312 039
CLADE ground truth	563.58h	54.80 h	1.01 h	7.57 m	619.51 h	44 664 231

Table S9: **Description of the simulated datasets with increasing percentages of eukaryotic sequences used for the analysis reported in Figure 6**

Dataset	Eukaryote CDS %	# CDS	Average length	# generated fragments	Fragment length
Euk10	10%	75 000	319 aa	717 879	50 aa
Euk20	20%		333 aa	748 269	
Euk30	30%		341 aa	767 927	
Euk40	40%	75 000	353 aa	793 350	50 aa
Euk50	50%		363 aa	816 661	
Euk60	60%		373 aa	838 736	
Euk70	70%	75 000	385 aa	865 560	50 aa
Euk80	80%		394 aa	887 109	
Euk90	90%		405 aa	910 946	

Note that the total length of the fragments in a dataset corresponds to 1.5 * #CDS * CDS_average_length.

Table S10: **Species and Models used to perform the analysis described in Figure 6**

**Domain**		**Species**	**Models**
Bacteria		3 128	1 397 356
Eukaryota	Metazoa	5 333	339 271
	Fungi	628	284 044
	Viridiplantae	2 260	100 085
	Alveolata	231	37 704
	Stramenopiles	72	27 616
	Euglenozoa	52	19 576
	Amoebozoa	19	16 186
	Others	808	34 426
Archaea		218	101 171
Viruses		2 127	31 799

Table S11: **Characteristics of the simulated marine MG dataset used to compare MetaCLADE and HMM-GRASPx.** The number of reads is related to the 100 bp dataset. In order to keep the same genome composition/coverage, the 200 bp dataset contains exactly the half of the reads.

RefSeq ID	Name	Genome size (bp)	Simulated reads	Coverage	%
NC_005070	Synechococcus sp. WH 8102	2 434 428	790 100	32.46X	4.04
NC_005071	Prochlorococcus marinus MIT9313	2 410 873	1 515 140	62.85X	7.75
NC_006370	Photobacterium profundum SS9 chr I	4 085 304	430 200	10.53X	2.20
NC_006371	Photobacterium profundum SS9 chr II	2 237 943	233 770	10.45X	1.20
NC_006576	Synechococcus elongatus PCC 6301	2 696 255	883 500	32.77X	4.52
NC_007205	Pelagibacter ubique HTCC1062	1 308 759	1 131 050	86.42X	5.79
NC_007484	Nitrosococcus oceani ATCC 19707	3 481 691	1 124 360	32.29X	5.75
NC_007516	Synechococcus sp. CC9605	2 510 659	806 700	32.13X	4.13
NC_007577	Prochlorococcus marinus str. MIT 9312	1 709 204	1 031 630	60.36X	5.28
NC_007722	Erythrobacter litoralis HTCC2594	3 052 398	340 890	11.17X	1.74
NC_007954	Shewanella denitrificans OS217	4 545 906	482 350	10.61X	2.47
NC_008209	Roseobacter denitrificans OCh 114	4 133 097	469 180	11.35X	2.40
NC_008319	Synechococcus sp. CC9311	2 606 748	839 940	32.22X	4.30
NC_008816	Prochlorococcus marinus str. AS9601	1 669 886	1 018 790	61.01X	5.21
NC_008817	Prochlorococcus marinus str. MIT 9515	1 704 176	1 018 290	59.75X	5.21
NC_009052	Shewanella baltica OS155	5 127 376	546 450	10.66X	2.80
NC_009091	Prochlorococcus marinus str. MIT 9301	1 641 879	1 002 710	61.07X	5.13
NC_009482	Synechococcus sp. RCC307	2 224 914	729 330	32.78X	3.73
NC_009783	Vibrio campbellii ATCC BAA-1116 chr I	3 765 351	410 040	10.89X	2.10
NC_009784	Vibrio campbellii ATCC BAA-1116 chr II	2 204 018	241 230	10.95X	1.23
NC_011138	Alteromonas mediterranea DE	4 480 937	479 070	10.69X	2.45
NC_011184	Vibrio fischeri MJ11 chr I	2 905 029	310 070	10.67X	1.59
NC_011186	Vibrio fischeri MJ11 chr II	1 418 848	149 900	10.56X	0.77
NC_013771	Atelocyanobacterium thalassa isolate ALOHA	1 443 806	411 620	28.51X	2.11
NC_014472	Maribacter sp. HTCC2170	3 868 304	2 070 810	53.53X	10.59
NC_015380	Pelagibacter sp. IMCC9063	1 284 727	1 082 970	84.30X	5.54

Table S12: **Pathway-family association of the simulated marine MG dataset used to compare MetaCLADE and HMM-GRASPx.**

**Pathway**	**Pfam domain family**
KO00010	PF00037 PF00044 PF00113 PF00121 PF00162 PF00294 PF00300 PF00370 PF00480 PF00501 PF00722 PF01112 PF01380 PF01408 PF01558 PF01676 PF01855 PF01950 PF02685 PF02775 PF02780 PF02800 PF03952 PF06013 PF07476 PF08543 PF09347 PF09741 PF10143 PF10432 PF12797 PF12798 PF12800 PF12837 PF12838 PF13183 PF13187 PF13193 PF13237 PF13292 PF13370 PF13378 PF13484 PF13534 PF13580 PF13746 PF14697
KO00020	PF00037 PF00056 PF00096 PF00118 PF00166 PF00180 PF00364 PF00529 PF00675 PF00682 PF00708 PF00814 PF01155 PF01210 PF01300 PF01363 PF01558 PF01597 PF01702 PF01855 PF02056 PF02254 PF02436 PF02492 PF02543 PF02737 PF02775 PF02780 PF02866 PF03446 PF03721 PF03807 PF04481 PF05193 PF05681 PF05683 PF05896 PF07503 PF07649 PF09723 PF09891 PF10571 PF11104 PF12700 PF12797 PF12798 PF12800 PF12837 PF12838 PF13183 PF13187 PF13237 PF13292 PF13370 PF13375 PF13437 PF13484 PF13533 PF13534 PF13717 PF13719 PF13738 PF13746 PF13805 PF13894 PF13912 PF14311 PF14446 PF14697
KO00030	PF00156 PF00215 PF00294 PF00456 PF00676 PF00834 PF00923 PF01380 PF01918 PF01950 PF02502 PF02581 PF02779 PF02780 PF06013 PF08543 PF10432 PF13292 PF13793 PF14272 PF14572
KO00051	PF00004 PF00005 PF00006 PF00037 PF00107 PF00121 PF00132 PF00290 PF00291 PF00294 PF00448 PF00483 PF00485 PF00596 PF00892 PF00931 PF00977 PF01027 PF01047 PF01078 PF01325 PF01380 PF01494 PF01656 PF01695 PF01726 PF01884 PF01950 PF01978 PF02082 PF02367 PF02463 PF02502 PF02511 PF02562 PF03169 PF03193 PF03205 PF03437 PF03564 PF03596 PF03631 PF03729 PF03739 PF03741 PF03965 PF05116 PF05729 PF06013 PF06193 PF06414 PF06738 PF07728 PF07907 PF08240 PF08282 PF08352 PF08477 PF08543 PF09488 PF09678 PF09906 PF10156 PF10432 PF10518 PF10662 PF11139 PF11143 PF12710 PF12793 PF12797 PF12800 PF12802 PF12804 PF12837 PF12838 PF12840 PF12846 PF13183 PF13187 PF13191 PF13207 PF13237 PF13238 PF13245 PF13304 PF13344 PF13346 PF13401 PF13412 PF13463 PF13476 PF13481 PF13484 PF13486 PF13520 PF13534 PF13545 PF13555 PF13601 PF13604 PF13614 PF13671 PF13886 PF13936 PF14150 PF14272 PF14602 PF14667 PF14691 PF14697
KO00620	PF00037 PF00056 PF00096 PF00118 PF00166 PF00364 PF00391 PF00501 PF00529 PF00675 PF00682 PF00708 PF00814 PF01155 PF01210 PF01300 PF01326 PF01363 PF01558 PF01597 PF01702 PF01855 PF02056 PF02254 PF02436 PF02492 PF02543 PF02737 PF02775 PF02780 PF02866 PF02896 PF03446 PF03721 PF03807 PF04055 PF04481 PF05193 PF05681 PF05683 PF05896 PF06451 PF07503 PF07649 PF08502 PF08726 PF09723 PF09741 PF09891 PF10571 PF11104 PF12700 PF12797 PF12798 PF12800 PF12837 PF12838 PF13183 PF13187 PF13193 PF13237 PF13292 PF13370 PF13375 PF13437 PF13484 PF13533 PF13534 PF13714 PF13717 PF13719 PF13738 PF13746 PF13805 PF13894 PF13912 PF14174 PF14311 PF14446 PF14697
KO00680	PF00005 PF00037 PF00056 PF00113 PF00155 PF00265 PF00266 PF00270 PF00294 PF00300 PF00384 PF00389 PF00391 PF00437 PF00448 PF00464 PF00501 PF00749 PF00910 PF00931 PF01041 PF01053 PF01210 PF01212 PF01326 PF01388 PF01478 PF01558 PF01568 PF01580 PF01637 PF01676 PF01750 PF01842 PF01855 PF01926 PF01935 PF01950 PF02056 PF02254 PF02463 PF02492 PF02562 PF02737 PF02742 PF02775 PF02780 PF02826 PF02866 PF02896 PF03029 PF03205 PF03266 PF03308 PF03446 PF03461 PF03599 PF03721 PF03807 PF03952 PF04055 PF04060 PF04879 PF04917 PF05116 PF05729 PF06271 PF06414 PF06451 PF06750 PF07476 PF07963 PF07991 PF08282 PF08433 PF08543 PF09488 PF09741 PF10143 PF10236 PF10412 PF10443 PF10518 PF11602 PF12675 PF12710 PF12797 PF12798 PF12800 PF12837 PF12838 PF12846 PF13086 PF13173 PF13183 PF13187 PF13191 PF13193 PF13207 PF13237 PF13238 PF13245 PF13247 PF13292 PF13344 PF13353 PF13370 PF13378 PF13394 PF13401 PF13459 PF13476 PF13481 PF13484 PF13534 PF13544 PF13555 PF13604 PF13633 PF13671 PF13738 PF13746 PF14691 PF14697
KO00910	PF00070 PF00120 PF00148 PF00163 PF00208 PF00411 PF00416 PF00444 PF00557 PF00572 PF00575 PF00717 PF00890 PF01000 PF01134 PF01176 PF01193 PF01196 PF01262 PF01266 PF01416 PF01479 PF01488 PF01494 PF01593 PF01946 PF02558 PF02826 PF03118 PF03486 PF03721 PF03807 PF03951 PF05833 PF07992 PF09954 PF10662 PF12396 PF12831 PF13187 PF13241 PF13275 PF13434 PF13450 PF13454 PF13738 PF14444 PF14520 PF14691
KO00920	PF00107 PF00132 PF00156 PF00291 PF06426 PF13561 PF14602

Table S13: **Comparison of HMM-GRASPx and MetaCLADE against a simulated 200-bp marine data set with uneven coverage.** MetaCLADE/HMM-GRASPx-Assembly is the annotation obtained by applying MetaCLADE on the assembled contigs of HMM-GRASPx and transferring it to the reads that map against annotated contigs. In the "strict" domain annotation table, only hits having the same pfam domain with respect to the ground-truth are counted as true positives. In the "clan-based" annotation, however, domain hits that belong to the same clan with respect to the ground-truth are also counted as true positives. The assembly of the KO00680 dataset would have required more than 128GB of RAM and we were not able to finish its construction.

"Strict" domain annotation

Pathway	HMM-GRASPx						MetaCLADE/HMM-GRASPx-Assembly						MetaCLADE/GC-Assembly					
	TP	FP	FN	TPR	PPV	F-score	TP	FP	FN	TPR	PPV	F-score	TP	FP	FN	TPR	PPV	F-score
KO00010	102 914	679	6 059	94.4	99.3	96.8	105 091	1 906	2 655	97.5	98.2	**97.9**	99 809	3 837	6 006	94.3	96.3	95.3
KO00020	125 427	5 098	12 271	91.1	96.1	93.5	129 020	10 174	3 602	97.3	92.7	**94.9**	120 393	14 618	7 785	93.9	89.2	91.5
KO00030	62 720	163	2 183	96.6	99.7	98.2	63 624	338	1 104	98.3	99.5	**98.9**	62 123	343	2 600	96.0	99.5	97.7
KO00051	298 382	8 435	30 671	90.7	97.3	**93.9**	301 317	25 554	10 617	96.6	92.2	**94.3**	289 664	21 691	26 133	91.7	93.0	92.4
KO00620	166 454	4 735	14 868	91.8	97.2	94.4	171 047	10 344	4 666	97.3	94.3	**95.8**	159 055	15 635	11 367	93.3	91.0	92.2
KO00680	*not available*						*not available*						372 002	37 724	35 283	91.3	90.8	**91.1**
KO00910	152 579	7 664	12 512	92.4	95.2	93.8	153 782	14 430	4 543	97.1	91.4	**94.2**	146 821	14 637	11 297	92.9	90.9	91.9
KO00920	47 984	12	9 021	84.2	100.0	91.4	49 800	287	6 930	87.8	99.4	93.2	51 274	496	5 247	90.7	99.0	**94.7**

"Clan-based" domain annotation

Pathway	HMM-GRASPx						MetaCLADE/HMM-GRASPx-Assembly						MetaCLADE/GC-Assembly					
	TP	FP	FN	TPR	PPV	F-score	TP	FP	FN	TPR	PPV	F-score	TP	FP	FN	TPR	PPV	F-score
KO00010	103 593	0	6 059	94.5	100.0	97.2	106 955	42	2 655	97.6	100.0	**98.8**	103 554	92	6 006	94.5	99.9	97.1
KO00020	130 525	0	12 271	91.4	100.0	95.5	139 186	8	3 602	97.5	100.0	**98.7**	134 934	77	7 785	94.5	99.9	97.2
KO00030	62 883	0	2 183	96.6	100.0	98.3	63 939	23	1 104	98.3	100.0	**99.1**	62 440	26	2 600	96.0	100.0	97.9
KO00051	306 817	0	30 671	90.9	100.0	95.2	326 789	82	10 617	96.9	100.0	**98.4**	311 141	214	26 133	92.3	99.9	95.9
KO00620	171 186	3	14 868	92.0	100.0	95.8	180 988	403	4 666	97.5	99.8	**98.6**	173 558	1 132	11 367	93.9	99.4	96.5
KO00680	*not available*						*not available*						404 693	5 033	35 283	92.0	98.8	**95.3**
KO00910	160 068	175	12 512	92.8	99.9	96.2	167 541	671	4 543	97.4	99.6	**98.5**	160 591	867	11 297	93.4	99.5	96.4
KO00920	47 996	0	9 021	84.2	100.0	91.4	50 060	27	6 930	87.8	99.9	93.5	51 746	24	5 247	90.8	100.0	**95.2**
